# Small Extracellular Vesicle (sEV) Uptake from Lung Adenocarcinoma and Squamous Cell Carcinoma Alters T-Cell Cytokine Expression and Modulates Protein Profiles in sEV Biogenesis

**DOI:** 10.3390/proteomes13020015

**Published:** 2025-04-23

**Authors:** Hafiza Padinharayil, Jinsu Varghese, Pulikkottil Raphael Varghese, Cornelia M. Wilson, Alex George

**Affiliations:** 1 Jubilee Mission Medical College & Research Institute, Thrissur 680005, Kerala, India; hafizaaficvd@gmail.com (H.P.); drprvarghese@gmail.com (P.R.V.); 2 Department of Zoology, St. Thomas College, Kozhencherry, Pathanamthitta 689641, Kerala, India; jinsuvarghese@gmail.com; 3 School of Psychology and Life Sciences, Canterbury Christ Church University, Kent CT1 1QU, UK

**Keywords:** lung adenocarcinoma, squamous cell carcinoma, T cells, sEVs, cross-treatment, RNA, protein, immune modulation

## Abstract

Background: Despite advances in immunotherapy, non-small-cell lung carcinoma (NSCLC)’s clinical success is limited, possibly due to substantial immunological alterations in advanced cancer patients. This study examines the immunomodulatory effects of sEVs derived from lung adenocarcinoma (ADC) and squamous cell carcinoma (SCC) on T cells. Methods: SEVs were isolated from lung cancer cell lines and Jurkat-E6.1. SEV size and morphology were analyzed by NTA and TEM, respectively, while Western blotting confirmed sEV markers. SEV uptake was assessed, followed by resazurin assay, RNA isolation, quantification, cDNA preparation, RT-PCR, nano LC-MS, and bioinformatic analysis, before and after treating Jurkat-E6.1 cells with sEVs from A549 and SKMES1. Results: Cancer-derived sEVs were efficiently internalized by immune cells, reducing T-cell viability. The real-time PCR analysis showed downregulation of KI67, BCL2, BAX, TNFA, IL6, TGFβ, and IL10, suggesting reduced proliferation, dysregulated apoptosis, and impaired inflammatory and immunosuppressive signaling, and the upregulation of GZMB and IL2 suggests retained cytotoxic potential but possibly dysfunctional T-cell activation. Proteomic analysis revealed 39 differentially abundant proteins (DAPs) in ADC-treated T cells and 276 in SCC-treated T cells, with 19 shared DAPs. Gene Ontology (GO) analysis of these DAPs highlighted processes such as sEV biogenesis, metabolic pathways, and regulatory functions, with ADC sEVs influencing NAD metabolism, ECM binding, and oxidoreductase activity, while SCC sEVs affected mRNA stability, amino acid metabolism, and cadherin binding. The cytoplasmic colocalization suggests the presence of these proteins in the cellular and extracellular lumen, indicating the potential of further release of these proteins in the vesicles by T cells. Conclusion: Lung cancer-derived sEVs regulate T-cell activities through immunoregulatory signaling. The molecular interactions between sEVs and immune cells can reveal novel tumor immune regulatory mechanisms and therapeutic targets.

## 1. Introduction

Among the most prevalent malignancies, lung cancer is the primary cause of cancer-related mortality globally. Non-small-cell lung cancer (NSCLC) includes adenocarcinoma (ADC), squamous-cell carcinoma (SCC), and large-cell carcinoma (80% of NSCLC cases). Small-cell lung cancer (SCLC) makes up the remaining 15% of cases [1]. The complex and dynamic immunological alterations taking place inside the tumor microenvironment are primarily responsible for the unsatisfactory clinical effectiveness in treating advanced non-small-cell lung cancer (NSCLC) despite advances in immunotherapy. The release of extracellular vesicles (EV), especially small extracellular vesicles (sEVs), which are known to alter immune cell function and accelerate tumor growth, is one such mechanism linked to immune evasion [2,3].

Exosomes range in size from 30 to 200 nm. They are found in all bodily fluids and are generated by all cell types through the endosome route. We refer to them as small extracellular vesicles (sEVs) rather than “exosomes” in accordance with minimal information for studies of EV (MISEV) recommendations [4,5,6,7,8]. SEVs are considered potential elements of liquid biopsy, as their molecular and genetic constituents are similar to that of their parent cells [9]. SEVs play a crucial mediating role in many facets of cell-to-cell communication, including those pertaining to causes of illness. Cancer cells produce and release cancer-derived sEVs in vast quantities [10,11,12]. It has been reported that T-cell dysfunction for tumor immunity can be caused by a range of immune suppressive signals carried by tumor-derived EVs [13].

On the other hand, human plasma has a diverse mixture of circulating sEVs from various organs, including immune cells [14,15]. In recent research, sEVs produced from cancer have been shown to have the ability to modify immunological responses, which might lead to immune suppression in the tumor setting. However, little is known about the precise function of sEVs from various NSCLC subtypes in modifying T-cell activity [16,17].

Successful cancer immunotherapy has long been thought to be hampered by tumors’ capacity to evade the host immune system [18]. Human tumors can suppress immune cell activity, particularly that of anti-tumor effector cells such as CD8+ and CD4+ T lymphocytes, NK cells, and dendritic cells [19]. It has been reported that after a short incubation time, the sera of cancer patients may inhibit the activities of normal activated T cells but not the sera of normal control samples [20]. Later, it was shown that sEVs, which resembled the parental tumor cells’ cell-surface membranes in molecular makeup, were responsible for this suppressive impact [20,21].

In this work, we examine the immunomodulatory effects on T-cell lineages of sEVs secreted from two different subtypes of lung cancer cell lines: SCC and ADC. We hope to clarify the molecular processes behind the connection between tumor-derived sEVs and T-cell line by isolating sEVs from lung cancer cell lines (A549 and SKMES1) and evaluating their effects on T-cell survival, gene expression, and proteomic profile. This study specifically aims to determine how sEV-mediated signaling modifies T-cell activity and adds to the immunomodulatory milieu that characterizes advanced lung cancer in vitro.

## 2. Materials and Methods

### 2.1. Adherent and Suspension Cell Culture

Human lung cancer cells A549 (CCL-185, lot no: 70047545) and SKMES1 (HTB-58, lot no: 70037644) were purchased from ATCC, Manassas, VA, USA, while Jurkat E6.1 was acquired from the National Centre for Cell Science (NCCS), Pune, India. Following verification of cell line purity using short tandem repeat (STR), ATCC cell lines were procured following approval of the material transfer agreement (MTA) and biosafety level (BSL). Cell line procured from NCCS was identified for its identity using STR at NCCS. AmpFISTR Identifier Plus PCR amplification kit (Applied Biosystems, Foster City, CA, USA) was used to amplify STR loci. The Applied Biosystems 3500 Genetic Analyzer (Thermo Fisher Scientific, Foster City, CA, USA) was used to process the cell line. Applied Biosystems’ Gene Mapper ID-X v1.5 software was used to analyze the data and guaranteed that the cell line purity fully matched with the ATCC STR profile database.

A549 cell line and SKMES1 were adherent cells that were cultured in Ham’s F-12K and EMEM medium, respectively. The media was supplemented with 10% (*v*/*v*) sEV-free serum and 1% pen strep, and the cultures were maintained at 37 °C with 5% CO_2_. RPMI medium mixed with 10% FBS and 1% pen strep was used to culture Jurkat E6.1 in suspension.

### 2.2. Isolation of sEVs Using Precipitation Method

The cells were cultured in a medium containing serum devoid of sEVs for a minimum of 2.5–3 doublings until they reached 90% confluency. After collecting the conditioned media, it was centrifuged at 2000 rpm for 5 min to remove any cell debris. It was then centrifuged at 5000 rpm for 20 min and filtered using a 0.22 μm filter. sEVs were precipitated using the ExoEnrich high performance sEV isolation kit (Catalog No. PEC50, Exocan Healthcare, Pune, India). Two rounds of sEV washing were conducted using phosphate buffer solution (PBS).

### 2.3. Nanoparticle Tracking Analysis

SEV samples were filtered through 0.22 μm filter membranes and diluted 10- to 100-fold in PBS to achieve a particle count of around 100 per frame for the measurement. The size distribution and concentration of isolated sEVs were determined using a Nanoparticle Tracking Analyser (Particle Metrix Zetaview-PMX 130-Mono laser, Bavaria, Germany). As advised by the manufacturer, the camera level (9–12) and detection threshold (2–6) were manually changed. Each identified particle’s mean square displacement was calculated from the video that was captured. The program then calculated the sphere-equivalent hydrodynamic radius and diffusion coefficient using the Stokes–Einstein equation.

### 2.4. Transmission Electron Microscopy

The sEV suspension was fixed using 4% paraformaldehyde (final concentration of 2%). A sample of around 35 μL fixed sEVs was put onto copper-mesh support film coated with carbon, and the film was sealed for two to five minutes. Then, the surplus solution was removed from the edge and left to sit on the pointed filter paper for approximately ten minutes. Uranyl acetate dye solution was dropped and left for ninety seconds after the support film had dried. After the surplus dye solution was absorbed, the support film was clamped onto the filter paper and allowed to dry for three hours in order to observe.

### 2.5. SEV Quantification and Treatment

The Pierce bicinchoninic acid assay (BCA) kit (Catalog No. 23225, Thermo Fisher Scientific, Waltham, MA, USA) was used to measure the protein content of sEVs. Protein standards (BSA) were used to make a standard curve by serial dilution. Reagents A and B were combined in a 50:1 ratio to create the working reagent. To allow for color development, sEV samples and standards were combined with 200 µL of the working reagent and incubated for 30 min at 37 °C. A microplate reader was used to measure the absorbance at 562 nm once it had cooled to room temperature. The protein concentrations were determined by comparing the absorbance values of the sEV samples to the standard curve. Background absorbance was subtracted using a blank. For the treatments using A549 and SKMES1 derived sEVs on T-cell lines, 50 μg/mL sEV was used to treat cancer cell-derived (A549, SKMES1) sEVs on T cells (Jurkat E6.1) at 70% confluency.

### 2.6. Resazurin Cell Viability Assay

The effect of sEVs on cell viability were measured using fluorescent cell viability assay. Jurkat E6.1 was cultured (0.3 × 10^5^ cells per mL) in 96-well suspension plates and treated using A549- and SKMES1-derived sEVs made up to 200 μL. The cells with sEVs were incubated at 37 °C with 5% CO_2_ for 32 h. Resazurin was added to each well with a final concentration of 44 μM and measured the relative fluorescence unit (RFU) using a series of 590 nm emission and 560 nm excitation filters using the Tecan microplate reader Infinite 200 pro. The experiment was performed in triplicate.

### 2.7. Labelling of Cancer-Derived sEVs

Isolated sEVs from A549 and SKMES1 were diluted to 50 µg/mL of protein in ultra-pure water. The staining process involved mixing diluent C with 1 mM PKH67 stock (green, origin, D0031) in the ratio 9:1. After that, PKH67 (Origin, D0031) in diluent C was mixed with purified sEVs in the ratio of 1:25. We incubated the sEVs at 2–8 °C for 15–30 min. The sEVs were then re-isolated using the ExoEnrich kit (Exocan Healthcare) so as to remove unbound PKH67 dye, followed by two rounds of pellet wash to remove maximum unbound dye.

### 2.8. SEV Uptake and Cell Labelling

The labelled sEVs were incubated with the T cells for 24 h at 37 °C and 5% CO_2_. The cells were fixed onto the coverslip using 3.75% paraformaldehyde in PBS for 15 min on ice., followed by three rounds of PBS wash. The cells were permeabilized using 0.5% Triton X-100 in PBS for 10 min at room temperature and three rounds of PBS wash. To stain the nucleus, Hoechst 33258, a blue dye dissolved in 200 μM DMSO, was incubated at 37 °C at room temperature for 10 min at the working concentration of 5:39 (Hoechst 33258: RPMI 1640 media).

For cytoplasmic staining, the cells on the coverslip were incubated with SF680R phalloidin (origin, CA1650) at the ratio 5:39 (phalloidin: RPMI 1640 media) followed by incubation at 37 °C at room temperature for 10 min, followed by three rounds of PBS wash.

### 2.9. Microscopy for Image Capture

The coverslips having fixed cells stained for sEVs, nucleus, and cytoplasm were mounted onto the slides and captured using Zeiss fluorescent microscope (Axio, Scope.A1) using the Isis software, version 6.3.

### 2.10. Western Blot

A Western blot was performed to characterize sEVs. Cells were obtained and lysed for 30 min using the whole proteinase inhibitor cocktail (Roche, Basel, Switzerland) in radio immunoprecipitation assay (RIPA) lysis buffer (50 mM Tris–HCl, pH 7.5, 150 mM NaCl, 0.25% sodium deoxycholate, 0.1% nonidet P-40, and 0.1% Triton X-100) at 4 °C for 30 min. The isolated sEVs were lysed using ExoLyseP (PEL-25P) (Exocan Healthcare) at 95 °C for 15 min. The supernatant was collected and quantified following centrifugation at 14,000× *g* for 10 min. The proteins were quantified using BCA assay kit (Thermo Fisher Scientific, Waltham, MA, USA), and 20 μg of protein was loaded for SDS-PAGE.

For the following SDS-PAGE, the cell lysates and sEV samples were denatured for 10 min at 100 °C after adding the loading buffer. The proteins were then transferred to the PVDF membrane for twenty minutes. Membranes were blocked for two hours at room temperature using 5% BSA in TBST, and then, they were incubated with primary antibodies at 1:500 dilution (CD9 (OPR1919), CD63 (OPR4255), and calnexin (OPR1830); origin) for an overnight period at 4 °C. Following four 5 min long TBST (TBS, 0.05% Tween-20) washes, the membranes were then incubated for two hours at room temperature with the secondary antibody (1:3000) (peroxidase-conjugated goat anti-rabbit IgG). Lastly, TBST (Himedia, Thane, India) was used to wash the membranes five times for ten minutes each time. The antibody-reactive bands were exposed on radiographic film by using enhanced chemiluminescence (ECL plus, Bio-Rad, Hercules, CA, USA).

### 2.11. RNA Isolation, cDNA Preparation

Total RNA was isolated from T cells and the treatment groups using TRI regent (Sigma Aldrich, Bavaria, Germany). Subsequently cDNA was synthesized using a High-Capacity cDNA Reverse Transcription Kit (Applied Biosystems).

### 2.12. Nucleic Acid Quality Control

In accordance with the manufacturer’s instructions, the RNA and DNA concentrations were measured using NanoQuant (Tecan, Männedorf, Switzerland). The Infinite200 NanoQuant plate reader was used to measure the absorbance ratios (260/280 and 260/230) in order to approximate the purity of the RNA and cDNA.

### 2.13. Real Time PCR

The expressions of IL10, TGFB1, BAX, GZMB, IL2, IL6, KI67, BCL2, TNFA, and GAPDH (reference gene) were assessed in the T cells and T cells treated with cancer-derived sEVs using real-time PCR (CFX Opus 96). The annealing temperatures for each target corresponding to the PCR conditions are given in Table 1. SYBR Green (origin) was used as the fluorochrome to record the Cq value. The PCR mixture constituted 2X real-time master mix (5 μL), forward primer (0.5 μL), reverse primer (0.5 μL), template (50 ng), and nuclease-free water to make up the final solution to 10 μL. The run conditions were initial denaturation at 95 °C for 5 min; PCR cycles (40 cycles); denaturation: 95 °C for 15 s; annealing temperature: Table 1; melt curve analysis from 65 °C to 95 °C. The experiment was performed in triplicate.

### 2.14. Whole Protein Extraction Using Rapigest

sEV was isolated from three different cultures of A549 and SKMES1. The isolated sEVs from the cultures were pooled independently for A549 and SKMES1 and applied on T cells. The experiment was performed in triplicate. The T cells and T cells treated with A549- and SKMES1-derived sEVs were harvested and pooled using centrifugation at 1000 rpm 5 min. The cell pellets were washed two times using 50 mM ammonium bicarbonate buffer by centrifugation at 1200 rpm 3 min. The washed pellets were treated with 0.5% Rapigest extraction buffer (1 mg Rapigest detergent, add 200 μL of 50 mM NH_4_HCO_3_/DNAse/Protease inhibitor), resuspended vigorously, and sonicated at room temperature for 10 min. The procedure was repeat3ed three times until the DNA was extracted, and then, two freeze–thaw cycles were applied using liquid nitrogen, and the debris was removed through ultracentrifugation at 14,000 rpm 10 min. The supernatant collected was stored at −80 °C.

### 2.15. Tryptic Digestion

The salt content in the protein extract was removed by Amicon Ultra 0.5 mL centrifugal filters with 3000MWCO (UFC500324, Millipore, MA, USA), and 50 mM ammonium bicarbonate (ABC) (pH 7.8) was the digestion buffer utilized in this investigation. Each sample included around 100 μg (100 µL, concentration: 1 µg/µL) of protein that was trypsin-digested in solution. Using 5 µL of 100 mM 1,4-dithiothreitol (DTT) in ammonium bicarbonate (CAS No: 3483-12-3, Sigma Aldrich), the samples were first reduced for 30 min at 60 °C. The samples were alkylated by incubating them for 30 min at room temperature in the dark with 5 μL of 200 mM iodoacetamide in ABC (CAS No: 144-48-9, Sigma Aldrich). MS-grade trypsin in ABC [Cat no: T6567, Sigma Aldrich] was used to digest the samples in a 1:25 ratio of trypsin (Cat no: T6567, Sigma Aldrich) to protein, where the protein concentration was estimated by BCA assay. The samples were then incubated for 17 h at room temperature, and the enzymatic process ceased with 1.0% formic acid after 20 min at room temperature, and 100 µg/100 µL protein, 5 µL DTT, 5 µL IAA, 10 µL trypsin (0.04 µg/µL), and 1 µL formic acid served as the whole reaction mixture. The supernatant was collected after centrifuging the digested peptide solution for 12 min at 14,000 rpm.

### 2.16. Liquid Chromatography–Mass Spectrometry (nLC-MS)

LC gradient conditions were the following: solvent A: 0.1% formic acid (FA) in water; solvent B: 0.1% formic acid in acetonitrile (ACN). EASY-Spray PepMap RSLC C18 100A° 2 µm column was used at 40 °C (Thermo Fisher Scientific, Waltham, MA, USA) in the Orbitrap Eclipse Fusion Tribid MS connected with Ultimate 3000 RSLC nano UHPLC system (Thermo Fisher Scientific, Waltham, MA, USA). Thermo Fisher Scientific’s Easy nLC 1200 equipment was used for reverse-phase chromatography to separate the peptides. In short, the C18 nano trap column was loaded with 1 µg of the peptide and allowed to equilibrate at a 5 µL/min flow rate. A linear gradient of 1 to 40% solvent B was used to elute the peptides from the column, EASY-spray PepMap RSLC C18 reversed-phase column, 2 μm and 75 μm × 500 mm, over 55.5 min at a flow rate of 250 nL/min. This was followed by a 7.5 min rinse with 80% solvent B. Each sample received two injections as duplicates.

The Orbitrap Eclipse Fusion Tribrid MS platform (Thermo Fisher Scientific, Waltham, MA, USA) was used to measure the spectra when it was in the positive ion mode. The MS parameters included a run duration of 145 min in the peptide application mode with NSI ion source type. MS scans with a 350–16,500 *m*/*z* scan range for precursor ions of charge states 2–7 were acquired on the Orbitrap detector in profile mode at a resolution of 120,000 as the standard automatic gain control (AGC) and a dynamic exclusion time of 6 s in a data-dependent mode with a 3 s cycle time. A ddMS^2^ scan was performed using ion trap detection in rapid scan mode, with HCD activation at a fixed normalized collision energy of 28%. The quadrupole isolation window was set to 1.6 *m*/*z*, and isolation offset was off. The AGC target for ddMS^2^ scans was set to standard, with maximum injection time set to auto and micro scans set to 1. The cycle time was maintained at 3 s between master scans.

Protein database uniprotkb_homo_sapiens_and_reviewed_tru_2024_02_15.fasta was used for protein search. The Thermo Scientific™ Proteome Discoverer™ was used to quantify the protein levels. The software workflow is attached as the Supplementary File. By setting the downstream analysis of the peptide spectra matches (PSM) and false-discovery rate (FDR) for both protein and peptide to 1% (strict) and 5% (relaxed), only unique peptides with high confidence were chosen for the final protein group identification. Abundance was calculated by integrating the area under the peak curve. The total abundance of all detected peptides at the mentioned FDR was used to normalize the abundance of each protein group. The label-free quantification approach was used to transfer the total median values for each distinct peptide ion abundance to the corresponding protein.

### 2.17. Bioinformatics and Pathway Enrichment Analysis

The bioinformatics analysis was performed for proteomics data obtained following LC-MS analysis of T cells and T cells treated with cancer-derived sEVs. All possible contaminants found during the MS analysis were eliminated before the differential abundance analysis (DAA). An additional filtering criterion was used to examine the peptide data across the duplicate injections. FDR < 0.05 was used to evaluate the peptides that were differentially abundant.

The uniprot symbols were added to the MS data using biomaRT library (org.Hs.eg.db) in the R software 4.5.0. The functional enrichment analysis was performed for the abundant proteins in the T cells and treatment groups. The scatter plots, marginal histograms, and heatmaps were created using the server SRPlot (https://www.bioinformatics.com.cn/en; accessed on 5 November 2024). The volcano plots were created by VolcanoseR (https://huygens.science.uva.nl/VolcaNoseR/; accessed on 5 November 2024). GO analysis and visualization were performed using ShinyGO version 0.81 (https://bioinformatics.sdstate.edu/go/; accessed on 12 November 2024) and SRPlot. The Venn diagram was created using Venny 2.1.0 (https://bioinfogp.cnb.csic.es/tools/venny/; accessed on 6 November 2024).

### 2.18. Statistical Analysis

Two-way ANOVA followed by Turkey’s test was performed for the group comparison of RNA expression between T cells, T cells treated with ADC-derived sEVs, and SCC-derived sEVs. The analysis was performed using GraphPad prism 9.0, and a *p*-value < 0.05 was considered statistically significant. For the MS data, log2FC was calculated for individual proteins, and the *p*-value was calculated using Student’s *t*-test. Fold change ≤ or ≥1 was considered decreased or increased abundance, respectively. FDR < 0.05 was considered statistically significant.

## 3. Results

### 3.1. Identification and Characterization of ADC and SCC-Derived sEVs

**Figure 1:**
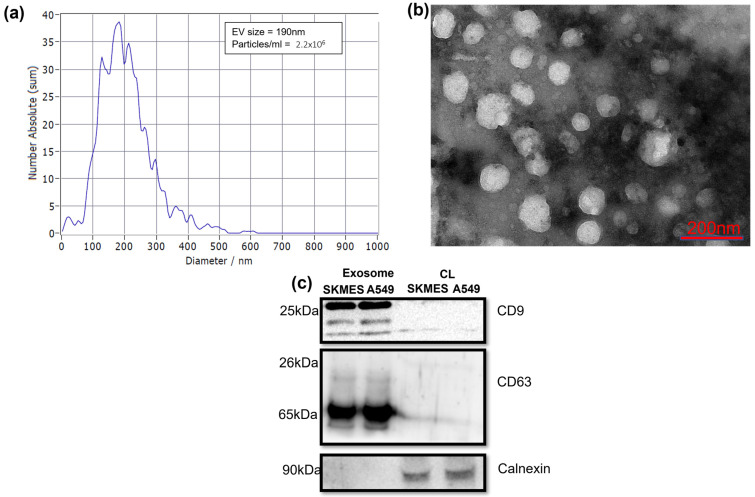
Characterization of sEV. (**a**) Nanoparticle tracking analysis (NTA) showing the size distribution and concentration of isolated small extracellular vesicles (sEVs) from A549 cells. (**b**) Transmission electron microscopy (TEM) analysis depicting the morphological characteristics of sEVs, with diameters ranging from 50 nm to 180 nm. Both the experiments were performed in triplicate. (**c**) Western blot analysis of sEV-specific markers: CD9 (25 kDa) and CD63 (25, 35–65 kDa), with calnexin (90 kDa) as a negative control. The first two lanes correspond to sEV samples, while the next two represent cell lysates. Abbreviations: EV, extracellular vesicle; CL, cell lysate.

SEVs were isolated from cell culture media of A549 and SKMES1 cells using the precipitation method. Nanoparticle tracking analysis (NTA) of the A549 cell line showed a particle concentration of 2.2 × 10^6^ per mL of culture media (Figure 1a and Supplementary Figure S1a) with a diameter range of 130 nm ± 20 nm. The structure of the isolate was characterized using transmission electron microscopy (TEM) (Figure 1b and Supplementary Figure S1b), and the spherical structure with a diameter ranging from 30 to 180 nm was observed. The vesicles were blotted against tetraspanins CD9 and CD63 (Figure 1c). The presence of CD9 and CD63 was unique to sEV samples, indicating the characteristic markers of sEVs. Calnexin was used to assess the cellular contamination, which was negligible in sEV samples. The observations from NTA, TEM, and immune blotting indicated the presence of sEVs in the isolates.

### 3.2. Cancer-Derived sEV Uptake by T Cells and Its Impact on Viability

**Figure 2:**
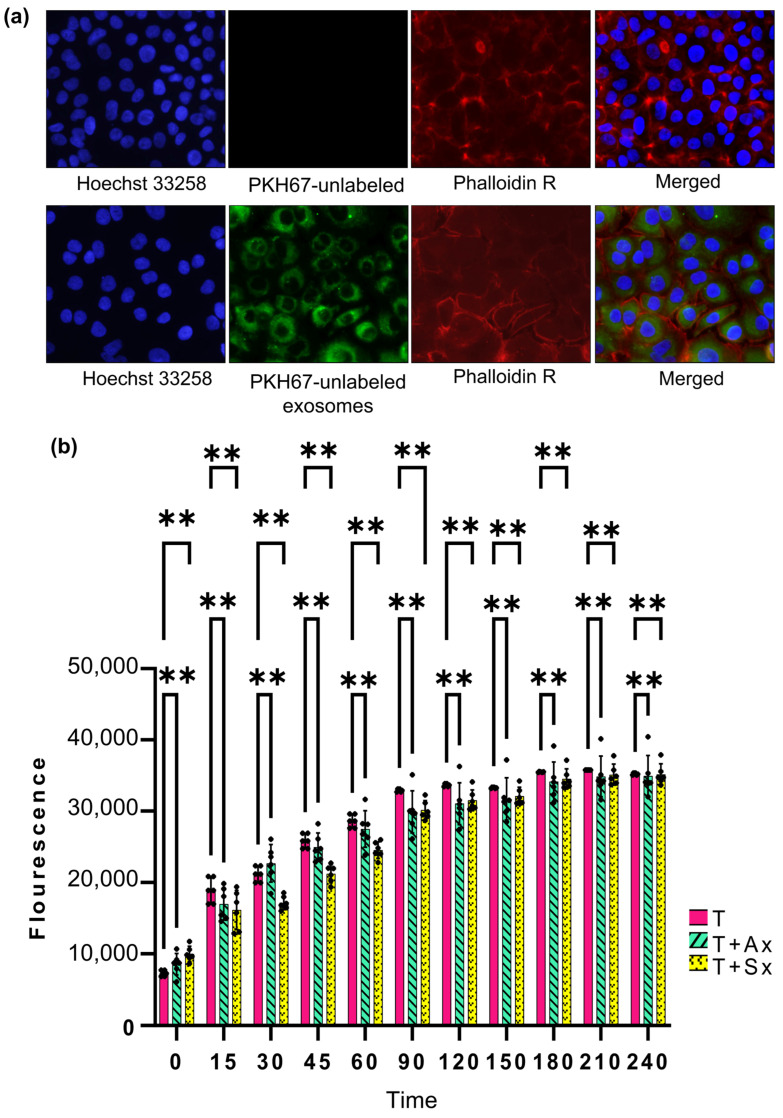
(**a**) Uptake of A549-derived small extracellular vesicles (sEVs) by T cells. sEVs are labeled in green, the nucleus in blue, and the plasma membrane in red. The presence of green fluorescence in the cytoplasm indicates the successful internalization of PKH-labeled sEVs. (**b**) Effect of cancer-derived sEVs on T-cell viability. T cells were treated with sEVs isolated from A549 and SKMES1 cells for 24 h, and viability was assessed using a resazurin assay. Fluorescence was recorded for the first four hours following resazurin addition. Abbreviations: T, Jurkat E6.1 cells; T + A549_Exo, Jurkat E6.1 cells treated with A549-derived sEVs; T + SKMES_Exo, Jurkat E6.1 cells treated with SKMES1-derived sEVs (the asterisks correspond to the *p*-value 0.0011).

We then treated T cells with PKH67 labeled cancer-derived sEVs (50 μg/mL) and kept them for 24 h under the appropriate growth conditions. The immune cells post incubation were fixed and stained with Hoechst nuclear dye and captured under fluorescent microscope (Figure 2a).

The resazurin viability assay was performed to assess the viability index of T cells upon A549 and SKEMS1-derived sEV treatment (Figure 2b). For this, we set up three groups: T cells, T cells treated with A549-derived sEVs (T_Ax), and T cells treated with SKMES1-derived sEVs (T_Sx). Resazurin assay was performed after 24 h of sEV incubation. The viability was high in the first two hours in the T cells relative to the T_Ax and T_Sx treatment groups but came to an equilibrium after two hours.

### 3.3. Comparative Analysis of Protein Abundance in T Cells Treated with ADC and SCC-Derived SEVs

**Figure 3:**
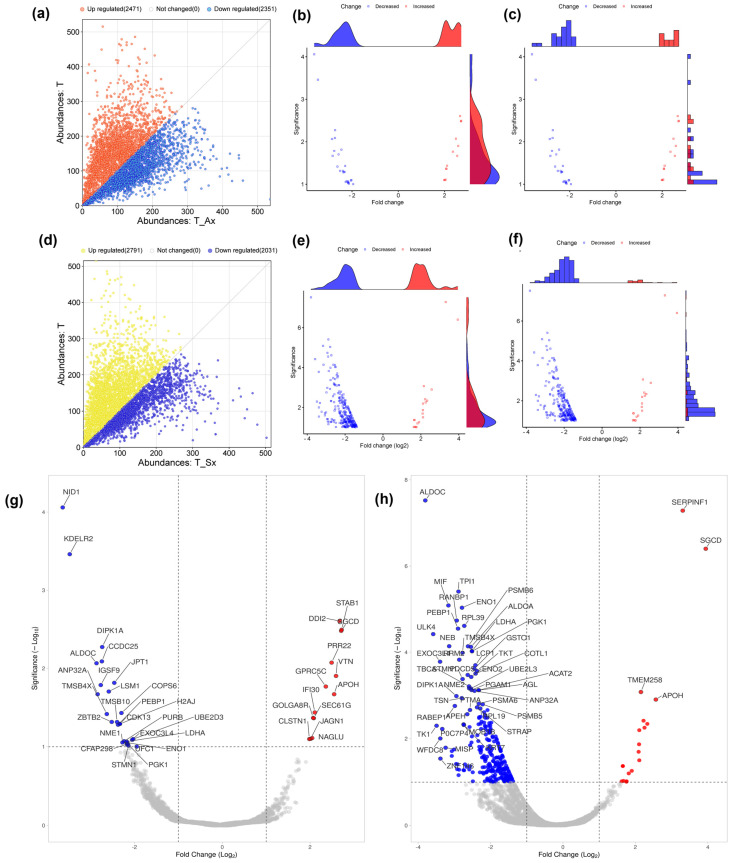
Comparison of T cells treated with ADC and SCC-derived sEVs in terms of protein abundance. (**a**) Scatterplot representing difference in the absolute abundance of T cells and T_Ax. (**b**) Marginal scatterplot with density diagram representing significantly abundant proteins between T cells and T_Ax treatment group. (**c**) Marginal bar plot representing significantly abundant proteins between T cells and T_Ax treatment group. (**d**) Scatterplot representing highly abundant and lower abundant proteins (DAP, logFC ± 1 and FDR < 0.05) in the T_Ax group. (**e**) Difference in the absolute abundance of T cells and T_Sx. (**f**) Marginal scatterplot with density diagram representing significantly expressed proteins between T cells and T_Sx treatment group. (**g**) Marginal bar plot representing significantly expressed proteins between T cells and T_Sx treatment group. (**h**) Volcano plot representing highly abundant and lower abundant proteins (DAP, logFC ± 1, and FDR < 0.05) in the T_Sx group.

The quantitative proteomic analysis of T cells and T cells treated with sEVs derived from ADC (T_Ax) and SCC (T_Sx) was performed using LC-MS orbitrap. The initial comparison using absolute protein abundance between T cells and T_Ax showed 2471 highly abundant proteins and 2351 low-abundant proteins (Figure 3a), while the comparison between T cells and T_Sx showed 2791 highly abundant and 2031 low-abundant proteins (Figure 3d). Absolute values were converted into fold change (log2FC), and *p*-values (log10p) were calculated using *t*-test. Proteins with ±1 FC and FDR < 0.05 were considered to trace out differentially abundant proteins (DAP) between the control and treatment groups.

Distribution of DAP between T cells and T_Ax (Figure 3b,c), between T cells and T_Sx (Figure 3e,f), and the best-hit fifty proteins are labeled in Figure 3g (T cell vs. T_Ax) and Figure 3h (T cell vs. T_Sx). In total, 39 DAP were traced in the T_Ax group (Supplementary Table S1) and 276 in T_Sx group (Supplementary Table S2). Nineteen proteins were common to both the treatment groups suggestive of a group of proteins altered by cancer-derived sEVs.

### 3.4. Gene Ontology of DAP Between T Cells and T Cells Treated with ADC sEVs

**Figure 4:**
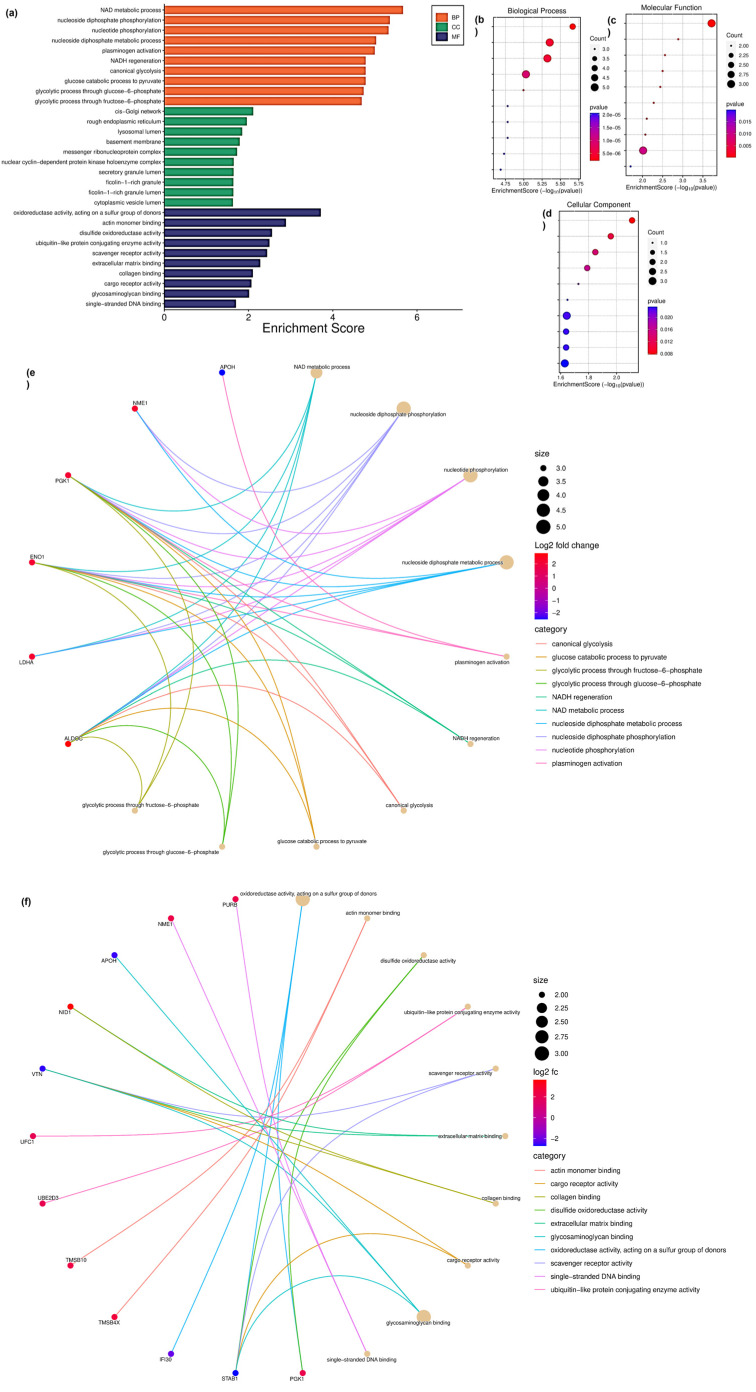
GO performed for DAP in the T cells treated with ADC-derived sEVs. (**a**) GO performed against enrichment score for biological processes (BP), molecular function (MF), and cellular compartmentalization (CC). (**b**) Enrichment bubble chart with enrichment score along with number of gene count in GO-BP. (**c**) Enrichment score along with number of gene count in GO-MF (**d**) Enrichment score along with number of gene count in GO-CC. (**e**) Cnetplot of the proteins involved in BP. (**f**) Cnetplot of the proteins involved in MF. The titles of BP and MF enrichment bubble chart are arranged accordingly to the GO bar chart.

The GO analysis performed using 39 DAP between T cells and the T_Ax group indicated the following: biological processes (BP), NAD metabolic process, nucleoside diphosphate phosphorylation and metabolism, nucleotide phosphorylation, plasminogen activation, NADH regeneration, and glycolysis (Figure 4a,b); molecular function (MF), oxidoreductase activity, actin monomer binding, ubiquitin-like protein activity, scavenger receptor activity, extracellular matrix (ECM) binding, collagen binding, cargo receptor activity, glycosaminoglycan binding, and ssDNA binding (Figure 4a,c); and cytoplasmic compartmentalization (CC), cis-Golgi, rough endoplasmic reticulum, lysosomal lumen, basement membrane, messenger ribonucleoprotein, secretory granule lumen, ficolin-1-rich granule and lumen, and cytoplasmic vesicle lumen (Figure 4a,d). The protein candidates involved in the corresponding GO mentioned above are represented in the cnetplot in Figure 4d,e.

### 3.5. Gene Ontology of DAP in T Cells and T Cells Treated with SCC sEVs

The GO analysis performed using 276 DAP between T cells and T_Sx group indicated the following processes: BP, proteasomal protein catabolic process (ubiquitin independent), mRNA catabolic processes and stability regulation, formation of cytoplasmic translation initiation complex, cytoplasmic translation, amino acid metabolic process, and planar polarity establishment (Supplementary Figure S2a,b); MF, threonine-type endopeptidase and peptidase activity, cadherin binding, translation regulation activity, unfolded protein binding, actin binding, and actin filament binding (Supplementary Figure S2a,c); and CC, ficolin-1-rich granule and lumen, proteasome core complex, cytoplasmic vesicle lumen, secretory granule lumen, eukaryotic 48s preinitiation complex, endopeptidase complex, and translation initiation factor 3 complex (Supplementary Figure S2a,d). The protein candidates involved in the corresponding GO mentioned are represented in the cnetplot in Supplementary Figure S2e,f.

### 3.6. Commonly Expressed Proteins upon T-Cell Treatment with ADC and SCC Derived sEVs

**Figure 5:**
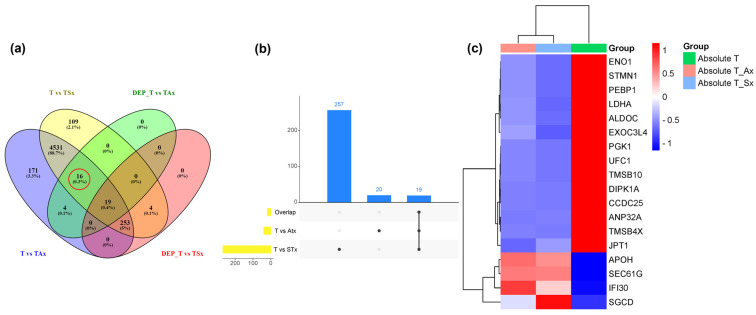
List of commonly expressed proteins in the T-cell treatment with ADC and SCC-derived sEVs. (**a**) Venn diagram showing number of commonly expressed proteins in various treatment groups. The red circle indicates 16 differentially abundant proteins (DAP) in T_Ax and T_Sx group. (**b**) Upset plot showing commonly expressed DAPs in T cells treated with A549-derived sEVs (T vs. ATx) and T cells treated with SKMES1-derived sEVs (T vs. STx). (**c**) Heatmap showing absolute abundance of nineteen DAP in T cells vs. T_Ax and T_Sx.

**Figure 6:**
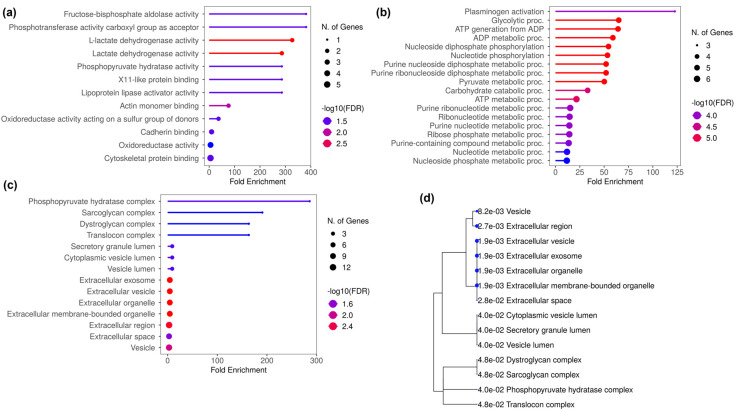
GO performed for the commonly expressed proteins in the T-cell treatment with ADC and SCC-derived sEVs. (**a**) GO performed against enrichment score for biological processes (BP). (**b**) Molecular function (MF). (**c**) Cellular compartmentalization (CC). (**d**) Tree map showing the cellular localization of DAP.

Nineteen proteins were common to DAP in T_Ax and T_Sx (Figure 5a–c). We then analyzed this intersection set to find whether these proteins possess any specific action on T cells. Interestingly, the GO of CC suggested the presence of these proteins in the cellular and extracellular lumen, indicating further release of these proteins in the vesicles by recipient cells (Figure 6c,d). The BP (Figure 6a) and MF (Figure 6b) of the intersection set was mostly linked to nucleic acid metabolism. The protein candidates involved in the corresponding GO mentioned above are represented in the cnetplot in Supplementary Figure S3a,b.

**Figure 7:**
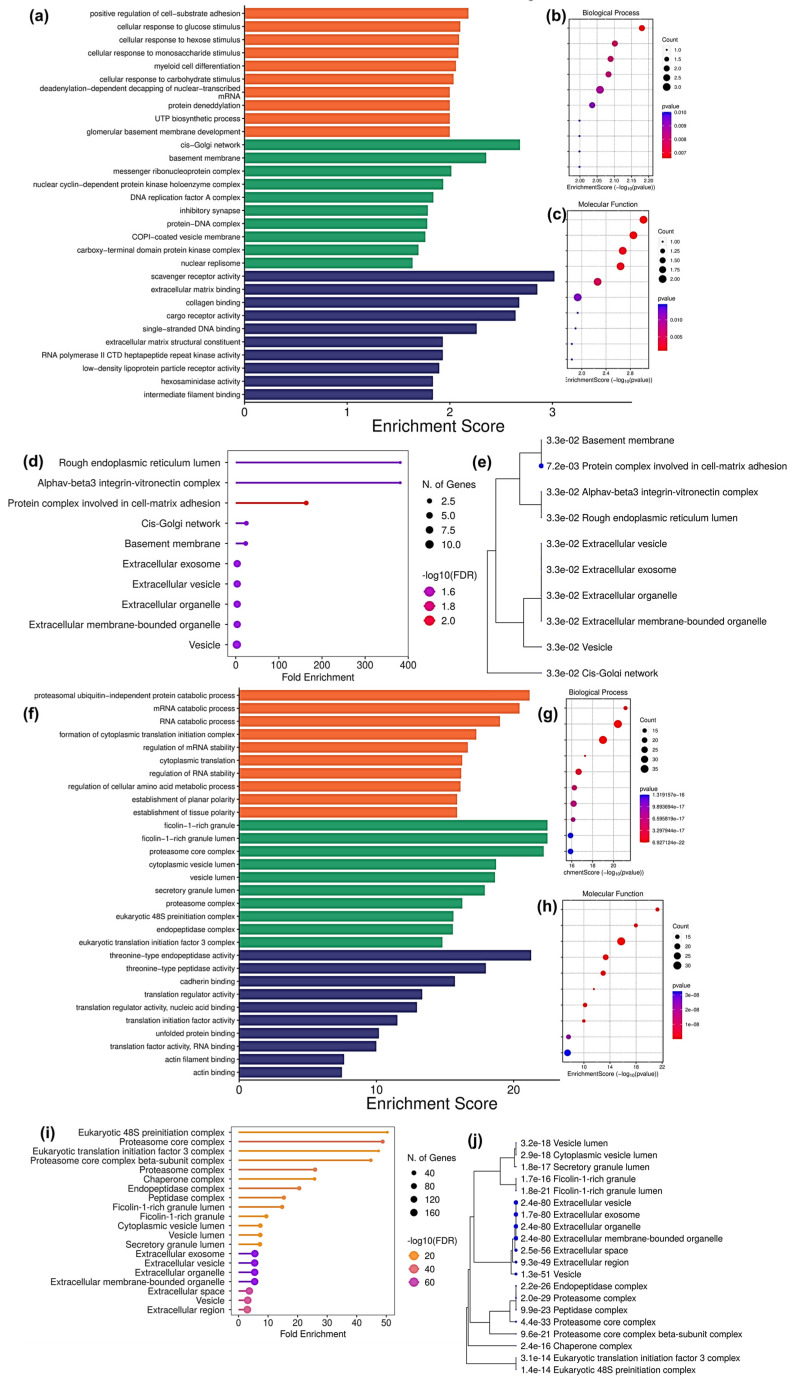
GO performed for DAP present uniquely to T_Ax and T_Sx. (**a**) GO performed for DAP uniquely expressed to T cells treated with ADC-derived sEVs. (**b**) GO-BP. (**c**) GO-MF. (**d**) ShinyGO CC enrichment. (**e**) ShinyGO CC enrichment tree map. (**f**) GO performed for DAP uniquely expressed to T cells treated with SCC-derived sEVs. (**g**) GO-BP. (**h**) GO-MF (**i**) ShinyGO CC enrichment. (**j**) ShinyGO CC enrichment tree map. The titles of BP and MF enrichment bubble chart are arranged accordingly to the GO bar chart.

Interestingly, the GO performed using DAP unique to T cells treated with ADC-derived sEVs (complement set of T_Ax vs. T_Ax Ո T_Sx) (Figure 7d,e and Supplementary Figure S4) and DAP unique to T cells treated with SCC derived sEVs (complement set of T_Sx vs. T_Ax Ո T_Sx) (Figure 7i,j and Supplementary Figure S5) also showed CC in the extracellular and vesicle lumen. The complement set of T_Sx vs. T_Ax Ո T_Sx also showed their presence in the ficolin-1-rich granule, cytoplasmic vesicle lumen, proteasome and chaperone complex, and in eukaryotic translation initiation complex. Other than the cytoplasmic and luminal presence, the complement set of T_Ax vs. T_Ax Ո T_Sx was localized at the cell–matrix adhesion, rough ER lumen, and cis-Golgi network.

Moreover, the GO performed for DAP unique to T_Ax showed the involvement in cell substrate adhesion, cellular response to glucose and monosaccharide stimulus, myeloid cell differentiation, protein deneddylation, and UTP biosynthetic process (Figure 7a,b) under BP as well as scavenger receptor activity, ECM binding, collagen binding, cargo receptor activity, ssDNA binding, RNA polymerase II CTD kinase activity, LDLR activity, and intermediate filament binding under MF (Figure 7a,c). Interestingly, DAP showed high potential to release as sEVs by T cells due to their localization in the cellular and extracellular compartments, which are involved in the sEV biogenesis pathway (Figure 6d and Figure 7e,j).

Additionally, the GO performed for DAP unique to T_Sx showed involvement in proteasomal ubiquitin-independent catabolic activity, RNA catabolic process, cytoplasmic translational initiation complex, mRNA and RNA stability regulation, amino acid metabolic process regulation, and the establishment of tissue planar polarity (Figure 7f,g) under BP as well as threonine-type endopeptidase and peptidase activity, cadherin binding, translation regulator binding, translation initiation factor activity, unfolded protein binding, RNA binding, and actin filament binding under MF (Figure 7f,h).

### 3.7. Cytokine Regulation in T Cells by Cancer-Derived sEVs

**Figure 8:**
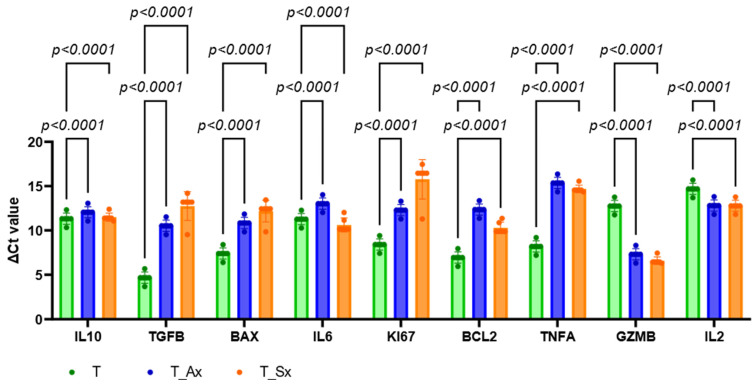
qPCR data showing the cytokine alteration and T-cell activity upon ADC and SCC-derived sEV treatment. Genes that are involved in the immunomodulation of T cells. The experiment was performed in triplicate.

The effects of A549- and SKMES1-derived small extracellular vesicles (sEVs) on T-cell activity were evaluated using real-time PCR. The mRNA levels of key immune-related and regulatory genes—IL10, TGFB, BAX, GZMB, Ki67, BCL2, IL6, IL2, and TNFA—were quantified (Figure 8 and Supplementary Figure S6). Both treatment groups exhibited significant downregulation of Ki67 (*p* < 0.0001), BCL2 (*p* < 0.0001), and BAX (*p* < 0.0001). Additionally, expression of inflammatory cytokines TNFA and IL6 was significantly reduced in both groups (*p* < 0.0001), although IL6 showed an increase in the T_Sx group compared to suppression in the T_Ax group. Immunosuppressive cytokines TGFβ and IL10 were significantly downregulated in both groups (*p* < 0.0001). Conversely, GZMB expression was upregulated in both treatment conditions. Increased levels of IL2 were also observed in both groups. These data suggest that exposure to lung cancer cell-derived sEVs alters T-cell gene expression patterns, particularly affecting pathways related to proliferation, apoptosis, and cytokine signaling.

## 4. Discussion

Although immunotherapeutic approaches have advanced significantly, there has not been much clinical success. This might be as a result of the severe immunological abnormalities discovered in people with advanced cancer. According to recent reports, tumor-derived sEVs cause T cells’ impaired signaling responses [21] by down-regulating Janus-activated kinase 3 and CD3, which triggers apoptosis [22]. Moreover, tumor sEVs may be able to specifically reduce lymphocytes’ responses to IL2 [23]. In light of these findings, we examined the function of lung cancer sEVs in immunomodulation. The present study demonstrates that Jurkat T cells are altered in the cytokine profile due to lung A549- and SKMES1-derived sEVs.

In the present study, the sEVs that were released by cancer cells were effectively up taken by immune cells. The resazurin assay was performed to assess the effects of sEV on the T-cell lines. Cancer-derived sEVs showed reduced T-cell viability in the first two hours of resazurin treatment but reached an equilibrium after two hours. The T cells showed alterations in the immunomodulation after A549- and SKMES1-derived exosome uptake. It has been reported that the exhausted T cells in the tumor microenvironment have a reduced capacity to generate cytokines like IL2 and lose their effector activities to regulate tumor development [24,25]. Treg cells can also develop from naive CD4+ T cells in the exposure of cancer-derived exosomes [26]. Immunosuppressive Treg cell differentiation may be induced by EVs produced from melanoma patients, but immune-active Th1 and Th17 lymphocyte differentiation may be hindered [27,28]. Conventional T cells are converted to CD4 + CD25highFoxP3+ Treg cells in a way that is reliant on TGFβ1 and IL10 since neutralizing antibodies specific to TGFβ1 and/or IL10 prevents tumor-derived EVs from proliferating Treg cells [27]. The real-time PCR results, where the changes in the expression of cytokines were used as the indicators of sEV effect on the T-cell line, indicate that cancer cell-derived exosomes significantly modulate T-cell function by altering key gene expression patterns, potentially contributing to immune suppression and tumor immune evasion. The observed downregulation of KI67 suggests reduced T-cell proliferation, while decreased expression of BCL2 and BAX indicates a dysregulated apoptotic balance, potentially predisposing the cells to AICD [29]. Furthermore, the suppression of TNFα and IL6 suggests a diminished inflammatory response, which may impair T-cell effector function and cytokine-mediated anti-tumor immunity [30]. The reduced expression of TGFβ and IL10 further implies a disruption in immunoregulatory signaling, which could compromise regulatory T-cell activity and immune homeostasis [31].

Interestingly, we found that GZMB and IL2 were upregulated in spite of these immunosuppressive effects, indicating a paradoxical rise in activation signals and cytotoxic potential. However, this activation can be transient and eventually result in T-cell dysfunction, exhaustion, or death in the absence of key survival signals like BCL2. These results are in line with previous studies showing that tumor-derived exosomes might influence immunological responses by promoting immunomodulatory pathways and reprogramming T-cell function [32,33]. SEV-mediated immune suppression may reduce the effectiveness of T-cell-based treatment approaches; hence, such alteration of T-cell function may have important effects on tumor growth and immunotherapy resistance [25]. To clarify the exact molecular processes by which cancer-derived exosomes orchestrate these immunological changes and investigate viable methods to reverse their immunosuppressive effects, more research is required.

The proteins that were distinguished between T cells and T cells treated with cancer-derived sEVs hint the role of cancer-derived sEVs on T cells. Our differential analysis on ADC and SCC-derived sEVs on T cells indicated 39 DAPs in ADC-derived sEV-treated T cells and 276 in SCC sEVs-treated cells, in which 19 proteins were common in both the groups, 20 proteins were unique to ADC treated group, and 257 proteins were unique to SCC-treated groups. Interestingly, upon GO analysis, these DAP showed high potential to release as sEVs by the recipient cells (T cells in our scenario) due to their localization in the cellular and extracellular compartments which are involved in the sEV biogenesis pathway (cytoplasmic luminal compartments, cis-Golgi network, etc.).

Crucially, the GO performed for DAP between T cells and T cells treated with ADC sEVs predicted their presence in the NAD metabolic process, nucleotide phosphorylation, oxidoreductase activity, extracellular matrix binding, ssDNA binding, scavenger receptor activity, etc. Furthermore, the GO performed for DAP between T cells and T cells treated with SCC sEVs predicted their presence in mRNA catabolic and stability regulation, translational regulation, the amino acid metabolic process, cellular polarity regulation, cadherin binding, unfolded protein binding, etc. Moreover, the GO performed using DAP unique to T cells treated with ADC-derived sEVs and DAP unique to T cells treated with SCC derived sEVs also showed CC in the extracellular and vesicle lumen. In addition, the DAP unique to squamous cell-derived sEV-treated T cells showed their presence in the ficolin-1-rich granule, cytoplasmic vesicle lumen, proteasome and chaperone complex, and in the eukaryotic translation initiation complex. Other than the cytoplasmic and luminal presence, the DAP unique to ADC cell-derived sEV-treated T cells were localized at the cell–matrix adhesion, rough ER lumen, and cis-Golgi network. Altogether, the results indicate the potential of these proteins to be trafficked along the endosomal pathways in T cells so as to release them in sEV form for the downstream cell–cell communication.

## 5. Conclusions

The current study found that lung cancer cells derived sEVs can induce immunomodulation on T cells. These sEVs modulate cytokine level in the T cells and their activity. The lung cancer-derived sEV induces DAP in the T cells, which are common and specific to the ADC and SCC histotypes. These DAP further possess the potential to be released by the T cells in sEV form. Further knowledge of the molecular processes underlying the interaction of immune cells with sEVs is also anticipated to yield important new information on the mechanism of tumor immune regulation.

## 6. Limitations and Future Prospective

Although we considered the protein abundance in the samples and not the post-translational modifications (PTMs) for the present study, bottom-up proteomics has a number of drawbacks that may limit a thorough description of protein diversity. One major problem is that when proteins are broken down into peptides during digestion, important information about full-length proteins and the PTM that go along with them is lost. This restricts the capacity to completely capture proteoforms, which might include isoforms, PTMs, and alternative splicing. Furthermore, it is frequently challenging to differentiate between protein isoforms with similar peptide sequences since they may produce identical peptides when digested. Certain PTMs may be identified using bottom-up proteomics; however, it can be difficult to determine the exact location and intricacy of PTM patterns, especially when they appear on several residues in the same peptide [34,35].

Here, we examined Jurkat E6.1 cell lines to examine the impact of sEVs produced from cancer on T cells. Specific T-cell subtypes (CD4+ or CD8+ T cells) derived from peripheral blood can be immune-sorted and experimentally examined for a more thorough examination of the immunological impact. Moreover, this study’s reliance on an in vitro model, which might not accurately mimic the complexity of entire biological systems, like those in vivo, is one of its shortcomings. Since cultured cells do not fully represent the physiological environment, it is still up for debate whether results from culture models can be applied to human biology. Additionally, the use of a precipitation-based method for sEV isolation, while convenient and widely adopted, may co-isolate non-vesicular extracellular particles (NVEPs), potentially impacting the purity of the sample, although the absence of exosome negative marker calnexin in the blot mitigates it. The qPCR results showed both upregulation and downregulation of inflammatory markers, reflecting the complex, context-dependent roles of exosomes. While this biological variability is relevant, it also limits the ability to draw definitive conclusions about directionality in immune modulation.

## Figures and Tables

**Table 1:** Annealing temperatures against each cDNA targets.

Sl No.	Target mRNA	Annealing Temperature	Primer_Forward (5′-3′)	Primer_Reverse (5′-3′)
1	TGFB1	65	TACCTGAACCCGTGTTGCTCTC	GTTGCTGAGGTATCGCCAGGAA
2	BAX	64.4	TCAGGATGCGTCCACCAAGAAG	TGTGTCCACGGCGGCAATCATC
3	TNFA	64.4	CTCTTCTGCCTGCTGCACTTTG	ATGGGCTACAGGCTTGTCACTC
4	IL10	63	TCTCCGAGATGCCTTCAGCAGA	TCAGACAAGGCTTGGCAACCCA
5	IL6	63	AGACAGCCACTCACCTCTTCAG	TTCTGCCAGTGCCTCTTTGCTG
6	GZMB	63	CGACAGTACCATTGAGTTGTGCG	TTCGTCCATAGGAGACAATGCCC
7	IL2	62.4	AGAACTCAAACCTCTGGAGGAAG	GCTGTCTCATCAGCATATTCACAC
8	GAPDH	60	GTCTCCTCTGACTTCAACAGCG	ACCACCCTGTTGCTGTAGCCAA
9	BCL2	55.7	ATCGCCCTGTGGATGACTGAGT	GCCAGGAGAAATCAAACAGAGGC
10	KI67	53.9	GGGCCAATCCTGTCGCTTAAT	GTTATGCGCTTGCGAACCT

## Data Availability

The mass spectrometry proteomics data have been deposited to the ProteomeXchange Consortium via the PRIDE repository with the dataset identifier PXD063051 and 10.6019/PXD063051.

## Supplementary Materials

The following supporting information can be downloaded at https://www.mdpi.com/article/10.3390/proteomes13020015/s1. Supplementary Figure S1: Nanoparticle tracking analysis (NTA) and transmission electron microscopy (TEM) images of exosomes secreted from SKMES1; Supplementary Figure S2: GO performed for differentially abundant proteins (DAP) in the T cells treated with SCC-derived sEVs; Supplementary Figure S3: Cnet plots for the commonly abundant proteins in the T cells treatment with ADC and SCC-derived sEVs; Supplementary Figure S4: Involvement of unique differentially abundant proteins (DAP) in the T cells treated with lung adenocarcinoma-derived sEVs; Supplementary Figure S5: Involvement of unique DAP in the T cells treated with lung squamous cell carcinoma-derived sEVs; Supplementary Figure S6: Ct values of genes that are involved in the immunomodulation of T cells. Supplementary Table S1: DAPs involved in T cells treated with lung adenocarcinoma-derived sEVs with fold change and *p*-value; Supplementary Table S2: DAPs involved in T cells treated with lung squamous cell carcinoma-derived sEVs group with fold change and *p*-value.

## Abbreviations

The following abbreviations are used in this manuscript:

NSCLC	non-small cell lung carcinoma
ADC	lung adenocarcinoma
SCC	lung squamous cell carcinoma
SCLC	small cell lung cancer
MISEV	minimal information for studies of extracellular vesicles
sEV	small extracellular vesicles
NTA	nanoparticle tracking analysis
TEM	transmission electron microscopy
AICD	activation-induced cell death
BCA	bicinchoninic acid assay
DAP	differentially abundant proteins
GO	gene ontology
sEV	small extracellular vesicles
LC-MS	liquid chromatography–mass spectrometry
T_Sx	SKMES1-derived sEVs
T_Ax	A549-derived sEVs
PTM	post-translational modifications
STR	short tandem repeat
MTA	material transfer agreement
NCCS	National Centre for Cell Science
BSL	biosafety level
RIPA	radio immunoprecipitation assay
DTT	1,4-dithiothreitol
ABC	ammonium bicarbonate
ACN	acetonitrile
PSM	peptide spectra matches
FDR	false-discovery rate
ECM	extracellular matrix
AGC	automatic gain control
CC	cytoplasmic compartmentalisation
BP	biological process
MF	molecular function
