# Supplementary material for: Small Extracellular Vesicle (sEV) Uptake from Lung Adenocarcinoma and Squamous Cell Carcinoma Alters T-Cell Cytokine Expression and Modulates Protein Profiles in sEV Biogenesis
Source: Proteomes. 2025 Apr 23;13(2):15. doi: 10.3390/proteomes13020015 (PMC12101295; doi:10.3390/proteomes13020015)
Supplement: Supplementary file 1 [file proteomes-13-00015-s001.zip › proteomes supplementary/MS Software workflow.docx]

================================================================================

Consensus Step : Workflow

================================================================================

Result name: 20240829_Hafeeza

Result file: D:\Project_monthly\2024\Augest\Dr Alex_Hafeeza\Dr Alex_Hafeeza_27082024\Hafeeza_Dr Alex\20240829_Hafeeza.pdResult

Description: Result filtered for high confident peptides, with enhanced peptide and protein annotations. Add FASTA file with common contaminants to the Protein Marker node. Quan abunaces are normalized to the same total peptide amount per channel and scaled, so that the average abunce per protein and peptide is 100.

Workflow based on template: CWF_Comprehensive_Enhanced Annotation_LFQ_and_Precursor_Quan

Creation date: 8/29/2024 6:05:02 PM

Created with Discoverer version: 3.0.0.757

------------------------------------------------------------------

The workflow tree:

------------------------------------------------------------------

|-(0) MSF Files

|-(10) Feature Mapper

|-(15) Precursor Ions Quantifier

|-(1) PSM Grouper

|-(2) Peptide Validator

|-(3) Peptide and Protein Filter

|-(4) Protein Scorer

|-(7) Protein FDR Validator

|-(5) Protein Grouping

|-(6) Peptide in Protein Annotation

|-(9) Protein Marker

|-(8) Protein Annotation

Post-processing nodes:

--------------------------------

|-(12) Result Statistics

|-(13) Display Settings

|-(14) Data Distributions

------------------------------------------------------------------

Processing node 0: MSF Files

------------------------------------------------------------------

1. Storage Settings:

- Spectra to Store: Identified or Quantified

- Feature Traces to Store: All

2. Merging of Identified Peptide and Proteins:

- Merge Mode: Globally by Search Engine Type

3. FASTA Title Line Display:

- Reported FASTA Title Lines: Best match

- Title Line Rule: standard

4. PSM Filters:

- Maximum Delta Cn: 0.05

- Maximum Rank: 0

- Maximum Delta Mass: 0 ppm

Hidden Parameters:

- MSF File(s): D:\Project_monthly\2024\Augest\Dr Alex_Hafeeza\Dr Alex_Hafeeza_27082024\Hafeeza_Dr Alex\20240829_Hafeeza.msf

------------------------------------------------------------------

Processing node 10: Feature Mapper

------------------------------------------------------------------

1. Chromatographic Alignment:

- Perform RT Alignment: True

- Maximum RT Shift [min]: 10

- Mass Tolerance: 10 ppm

- Parameter Tuning: Coarse

2. Feature Linking and Mapping:

- RT Tolerance [min]: 0

- Mass Tolerance: 0 ppm

- Min. S/N Threshold: 5

------------------------------------------------------------------

Processing node 15: Precursor Ions Quantifier

------------------------------------------------------------------

1. General Quantification Settings:

- Peptides to Use: Unique + Razor

- Consider Protein Groups for Peptide Uniqueness: True

- Use Shared Quan Results: False

- Reject Quan Results with Missing Channels: False

2. Precursor Quantification:

- Precursor Abundance Based On: Intensity

- Min. # Replicate Features [%]: 0

3. Normalization and Scaling:

- Normalization Mode: Total Peptide Amount

- Scaling Mode: On All Average

4. Exclude Peptides from Protein Quantification:

- For Normalization: Use All Peptides

- For Protein Roll-Up: Use All Peptides

- For Pairwise Ratios: Exclude Modified

5. Quan Rollup and Hypothesis Testing:

- Protein Abundance Calculation: Summed Abundances

- N for Top N: 3

- Protein Ratio Calculation: Pairwise Ratio Based

- Maximum Allowed Fold Change: 100

- Imputation Mode: None

- Hypothesis Test: t-test (Background Based)

6. Quan Ratio Distributions:

- 1st Fold Change Threshold: 2

- 2nd Fold Change Threshold: 4

- 3rd Fold Change Threshold: 6

- 4th Fold Change Threshold: 8

- 5th Fold Change Threshold: 10

------------------------------------------------------------------

Processing node 1: PSM Grouper

------------------------------------------------------------------

1. Peptide Group Modifications:

- Site Probability Threshold: 75

------------------------------------------------------------------

Processing node 2: Peptide Validator

------------------------------------------------------------------

1. General Validation Settings:

- Validation Mode: Automatic (Control peptide level error rate if possible)

- Target FDR (Strict) for PSMs: 0.01

- Target FDR (Relaxed) for PSMs: 0.05

- Target FDR (Strict) for Peptides: 0.01

- Target FDR (Relaxed) for Peptides: 0.05

2. Specific Validation Settings:

- Validation Based on: q-Value

- Target/Decoy Selection for PSM Level FDR Calculation Based on Score: Automatic

- Reset Confidences for Nodes without Decoy Search (Fixed score thresholds): False

------------------------------------------------------------------

Processing node 3: Peptide and Protein Filter

------------------------------------------------------------------

1. Peptide Filters:

- Peptide Confidence At Least: High

- Keep Lower Confident PSMs: False

- Minimum Peptide Length: 6

- Remove Peptides Without Protein Reference: False

2. Protein Filters:

- Minimum Number of Peptide Sequences: 1

- Count Only Rank 1 Peptides: False

- Count Peptides Only for Top Scored Protein: False

------------------------------------------------------------------

Processing node 4: Protein Scorer

------------------------------------------------------------------

No parameters

------------------------------------------------------------------

Processing node 7: Protein FDR Validator

------------------------------------------------------------------

1. Confidence Thresholds:

- Target FDR (Strict): 0.01

- Target FDR (Relaxed): 0.05

------------------------------------------------------------------

Processing node 5: Protein Grouping

------------------------------------------------------------------

1. Protein Grouping:

- Apply strict parsimony principle: True

------------------------------------------------------------------

Processing node 6: Peptide in Protein Annotation

------------------------------------------------------------------

1. Flanking Residues:

- Annotate Flanking Residues of the Peptide: True

- Number Flanking Residues in Connection Tables: 1

2. Modifications in Peptide:

- Protein Modifications Reported: Only for Master Proteins

3. Modifications in Protein:

- Modification Sites Reported: All And Specific

- Minimum PSM Confidence: High

- Report Only PTMs: True

4. Positions in Protein:

- Protein Positions for Peptides: Only for Master Proteins

------------------------------------------------------------------

Processing node 9: Protein Marker

------------------------------------------------------------------

1. Contaminant Database:

- Protein Database: PD_Contaminants_2015_5.fasta

5. Annotate Species:

- As Species Map: False

- As Species Names: False

6. Mark Additional Entities:

- Annotation Groups: False

- Pathway Groups: False

- Modification Sites: True

- Peptide Isoform Groups: True

------------------------------------------------------------------

Processing node 8: Protein Annotation

------------------------------------------------------------------

1. Annotation Aspects:

- 1. Aspect: Biological Process

- 2. Aspect: Cellular Component

- 3. Aspect: Molecular Function

- 4. Aspect: None

- 5. Aspect: None

- 6. Aspect: None

2. Annotation/Pathway Groups:

- Protein Database: uniprotkb_HOMO_SAPIENS_AND_reviewed_tru_2024_02_15.fasta

------------------------------------------------------------------

Processing node 12: Result Statistics

------------------------------------------------------------------

No parameters

------------------------------------------------------------------

Processing node 13: Display Settings

------------------------------------------------------------------

1. General:

- Filter Set:

### Filter Set MasterProteinFilter.filterset contains the following filters:

### Row Filter for TargetProtein:

### Master is equal to Master

###

'magellan filter set' 1 'MasterProteinFilter.filterset' FiltersetProperties 1 'LastFileName' 'C:\Users\frank.berg\Desktop\MasterProteinFilter.filterset' Filter 'TargetProtein' 1 NARY_AND 1 = FilterConditionProperties 1 'NamedComparableFilterCondition/DisplayPropertyHint' 'Master' property 'Thermo.PD.EntityDataFramework.MasterProteinAssessment, Thermo.Magellan.EntityDataFramework' 'IsMasterProtein' constant 'Thermo.PD.EntityDataFramework.MasterProteinAssessment, Thermo.Magellan.EntityDataFramework' 'IsMasterProtein'

------------------------------------------------------------------

Processing node 14: Data Distributions

------------------------------------------------------------------

1. ID Distributions (Bottom-up):

- Peptides to Use: Only unique peptides based on protein groups

------------------------------------------------------------------

Workflow messages:

------------------------------------------------------------------

08/30/2024 06:34 AM Job Execution: Processing D:\Project_monthly\2024\Augest\Dr Alex_Hafeeza\Dr Alex_Hafeeza_27082024\Hafeeza_Dr Alex\20240829_Hafeeza.pdResult

08/30/2024 06:34 AM (0) MSF Files: D:\Project_monthly\2024\Augest\Dr Alex_Hafeeza\Dr Alex_Hafeeza_27082024\Hafeeza_Dr Alex\20240829_Hafeeza.msf

08/30/2024 06:34 AM (0) MSF Files: All 1 files are ready for processing.

08/30/2024 09:18 AM Job Execution: Finished D:\Project_monthly\2024\Augest\Dr Alex_Hafeeza\Dr Alex_Hafeeza_27082024\Hafeeza_Dr Alex\20240829_Hafeeza.pdResult

================================================================================

Processing Step A: Workflow

================================================================================

Result name: 20240829_Hafeeza

Result file: D:\Project_monthly\2024\Augest\Dr Alex_Hafeeza\Dr Alex_Hafeeza_27082024\Hafeeza_Dr Alex\20240829_Hafeeza.msf

Description: Processing workflow for precursor-based quantification. Ion trap-detected HCD or CID spectra using SequestHT with Percolator validation. Specify the FASTA database, labels used, and any additional modifications.

Workflow based on template: PWF_Tribrid_Precursor_Quan_and_LFQ_ITHCD_CID_SequestHT_Percolator

Creation date: 8/29/2024 6:04:59 PM

Created with Discoverer version: 3.0.0.757

------------------------------------------------------------------

The workflow tree:

------------------------------------------------------------------

|-(0) Spectrum Files RC

|-(1) Minora Feature Detector

|-(2) Spectrum Selector

|-(3) Sequest HT

|-(4) Percolator

------------------------------------------------------------------

Processing node 0: Spectrum Files RC

------------------------------------------------------------------

1. Search Settings:

- File Name(s) (Hidden):

C:\Users\Admin\Desktop\Raw Files\Dr Alex_Hafeeza\A_01.raw

C:\Users\Admin\Desktop\Raw Files\Dr Alex_Hafeeza\A_02.raw

C:\Users\Admin\Desktop\Raw Files\Dr Alex_Hafeeza\A+Tx_01.raw

C:\Users\Admin\Desktop\Raw Files\Dr Alex_Hafeeza\A+Tx_02.raw

C:\Users\Admin\Desktop\Raw Files\Dr Alex_Hafeeza\S_01.raw

C:\Users\Admin\Desktop\Raw Files\Dr Alex_Hafeeza\S_02.raw

C:\Users\Admin\Desktop\Raw Files\Dr Alex_Hafeeza\S+Tx_01.raw

C:\Users\Admin\Desktop\Raw Files\Dr Alex_Hafeeza\S+Tx_02.raw

C:\Users\Admin\Desktop\Raw Files\Dr Alex_Hafeeza\T_01.raw

C:\Users\Admin\Desktop\Raw Files\Dr Alex_Hafeeza\T_02.raw

C:\Users\Admin\Desktop\Raw Files\Dr Alex_Hafeeza\T+Ax_01.raw

C:\Users\Admin\Desktop\Raw Files\Dr Alex_Hafeeza\T+Ax_02.raw

C:\Users\Admin\Desktop\Raw Files\Dr Alex_Hafeeza\T+Sx_01.raw

C:\Users\Admin\Desktop\Raw Files\Dr Alex_Hafeeza\T+Sx_02.raw

- Protein Database: uniprotkb_HOMO_SAPIENS_AND_reviewed_tru_2024_02_15.fasta

- Enzyme Name: Trypsin (Full)

- Precursor Mass Tolerance: 20 ppm

- Fragment Mass Tolerance: 0.5 Da

- 1. Static Modification: Carbamidomethyl / +57.021 Da (C)

2. Regression Settings:

- Regression Model: Non-linear Regression

- Parameter Tuning: Coarse

------------------------------------------------------------------

Processing node 1: Minora Feature Detector

------------------------------------------------------------------

1. Peak & Feature Detection:

- Min. Trace Length: 5

- S/N Threshold: 1

- Max. ΔRT of Isotope Pattern Multiplets [min]: 0.2

2. Feature to ID Linking:

- PSM Confidence At Least: High

------------------------------------------------------------------

Processing node 2: Spectrum Selector

------------------------------------------------------------------

1. General Settings:

- Precursor Selection: Use MS1 Precursor

- Use Isotope Pattern in Precursor Reevaluation: True

- Provide Profile Spectra: Automatic

2. Spectrum Properties Filter:

- Lower RT Limit: 0

- Upper RT Limit: 0

- First Scan: 0

- Last Scan: 0

- Lowest Charge State: 0

- Highest Charge State: 0

- Min. Precursor Mass: 350 Da

- Max. Precursor Mass: 5000 Da

- Total Intensity Threshold: 0

- Minimum Peak Count: 1

3. Scan Event Filters:

- MS Order: Is Not MS1

- Min. Collision Energy: 0

- Max. Collision Energy: 1000

- Scan Type: Is Full

4. Peak Filters:

- S/N Threshold (FT-only): 1.5

5. Replacements for Unrecognized Properties:

- Unrecognized Charge Replacements: Automatic

- Unrecognized Mass Analyzer Replacements: ITMS

- Unrecognized MS Order Replacements: MS2

- Unrecognized Activation Type Replacements: CID

- Unrecognized Polarity Replacements: +

- Unrecognized MS Resolution@200 Replacements: 60000

- Unrecognized MSn Resolution@200 Replacements: 30000

6. Precursor Pattern Extraction:

- Precursor Clipping Range Before: 2.5 Da

- Precursor Clipping Range After: 5.5 Da

------------------------------------------------------------------

Processing node 3: Sequest HT

------------------------------------------------------------------

1. Input Data:

- Protein Database: uniprotkb_HOMO_SAPIENS_AND_reviewed_tru_2024_02_15.fasta

- Enzyme Name: Trypsin (Full)

- Max. Missed Cleavage Sites: 2

- Min. Peptide Length: 6

- Max. Peptide Length: 144

- Max. Number of Peptides Reported: 10

2. Tolerances:

- Precursor Mass Tolerance: 10 ppm

- Fragment Mass Tolerance: 0.6 Da

- Use Average Precursor Mass: False

- Use Average Fragment Mass: False

3. Spectrum Matching:

- Use Neutral Loss a Ions: True

- Use Neutral Loss b Ions: True

- Use Neutral Loss y Ions: True

- Use Flanking Ions: True

- Weight of a Ions: 0

- Weight of b Ions: 1

- Weight of c Ions: 0

- Weight of x Ions: 0

- Weight of y Ions: 1

- Weight of z Ions: 0

4. Dynamic Modifications:

- Max. Equal Modifications Per Peptide: 3

- Max. Dynamic Modifications Per Peptide: 4

- 1. Dynamic Modification: Oxidation / +15.995 Da (M)

6. Dynamic Modifications (protein terminus):

- 1. N-Terminal Modification: Acetyl / +42.011 Da (N-Terminus)

7. Static Modifications:

- 1. Static Modification: Carbamidomethyl / +57.021 Da (C)

------------------------------------------------------------------

Processing node 4: Percolator

------------------------------------------------------------------

1. Target/Decoy Strategy:

- Target/Decoy Selection: Concatenated

- Validation based on: q-Value

2. Input Data:

- Maximum Delta Cn: 0.05

- Maximum Rank: 0

3. FDR Targets:

- Target FDR (Strict): 0.01

- Target FDR (Relaxed): 0.05
