# Supplementary material for: Small Extracellular Vesicle (sEV) Uptake from Lung Adenocarcinoma and Squamous Cell Carcinoma Alters T-Cell Cytokine Expression and Modulates Protein Profiles in sEV Biogenesis
Source: Proteomes. 2025 Apr 23;13(2):15. doi: 10.3390/proteomes13020015 (PMC12101295; doi:10.3390/proteomes13020015)
Supplement: Supplementary file 1 [file proteomes-13-00015-s001.zip › proteomes supplementary/proteomes_supplementary file.docx]

Involvement of differentially abundant proteins (DAP) on the T cells treated with lung adeno and squamous cell carcinoma-derived sEV.

Appendix A: Supplementary figures


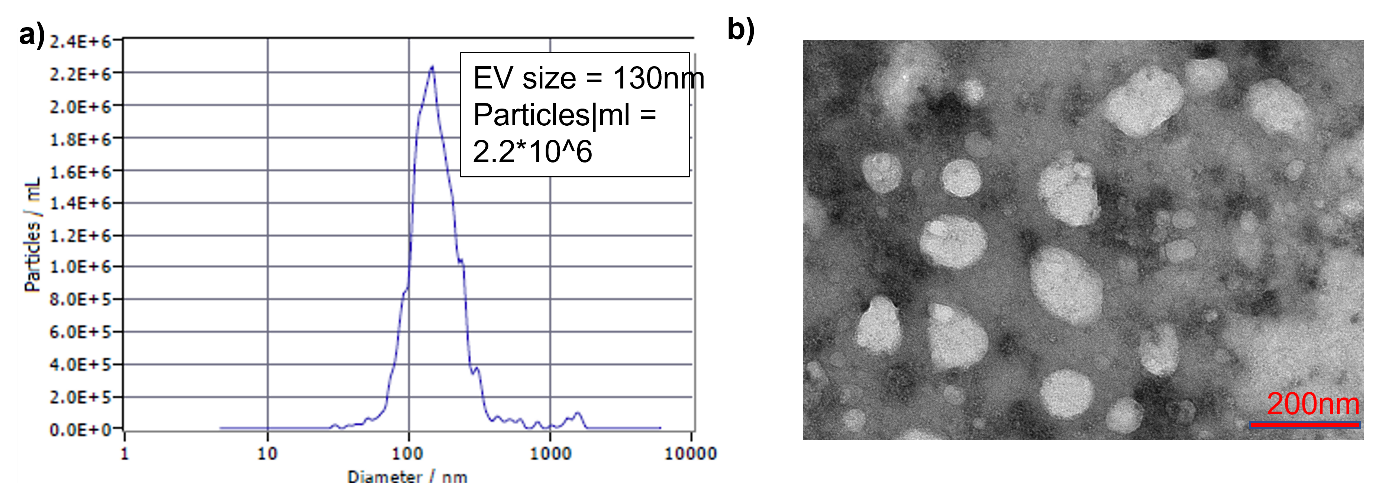


Supplementary figure 1: Supplementary figure 1: a) Nanoparticle tracking analysis (NTA) and b) transmission electron microscopy (TEM) images of exosomes secreted from SKMES1.


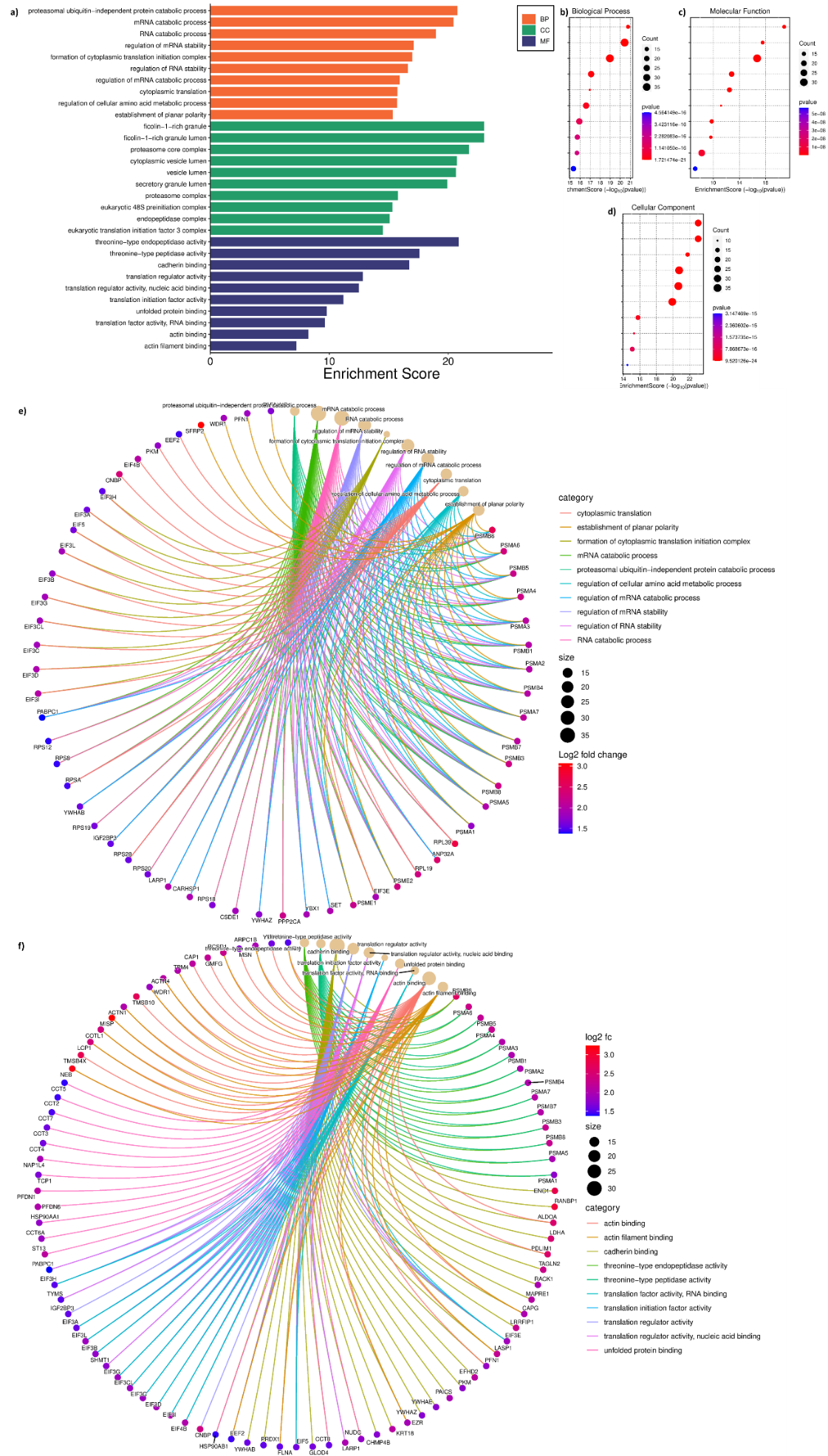


**Supplementary figure 2: GO performed for differentially abundant proteins (DAP) in the T cells treated with SCC-derived sEVs. a)** GO performed against enrichment score for biological processes (BP), molecular function (MF), and cellular compartmentalisation (CC). **b)** Enrichment score along with number of gene count in GO-BP **c)** Enrichment score along with number of gene count in GO-MF **d)** Enrichment score along with number of gene count in GO-CC **e)** cnetplot of the proteins involved in BP **f)** cnetplot of the proteins involved in MF. BP and MF enrichment bubble chart titles are arranged according to the GO bar chart.


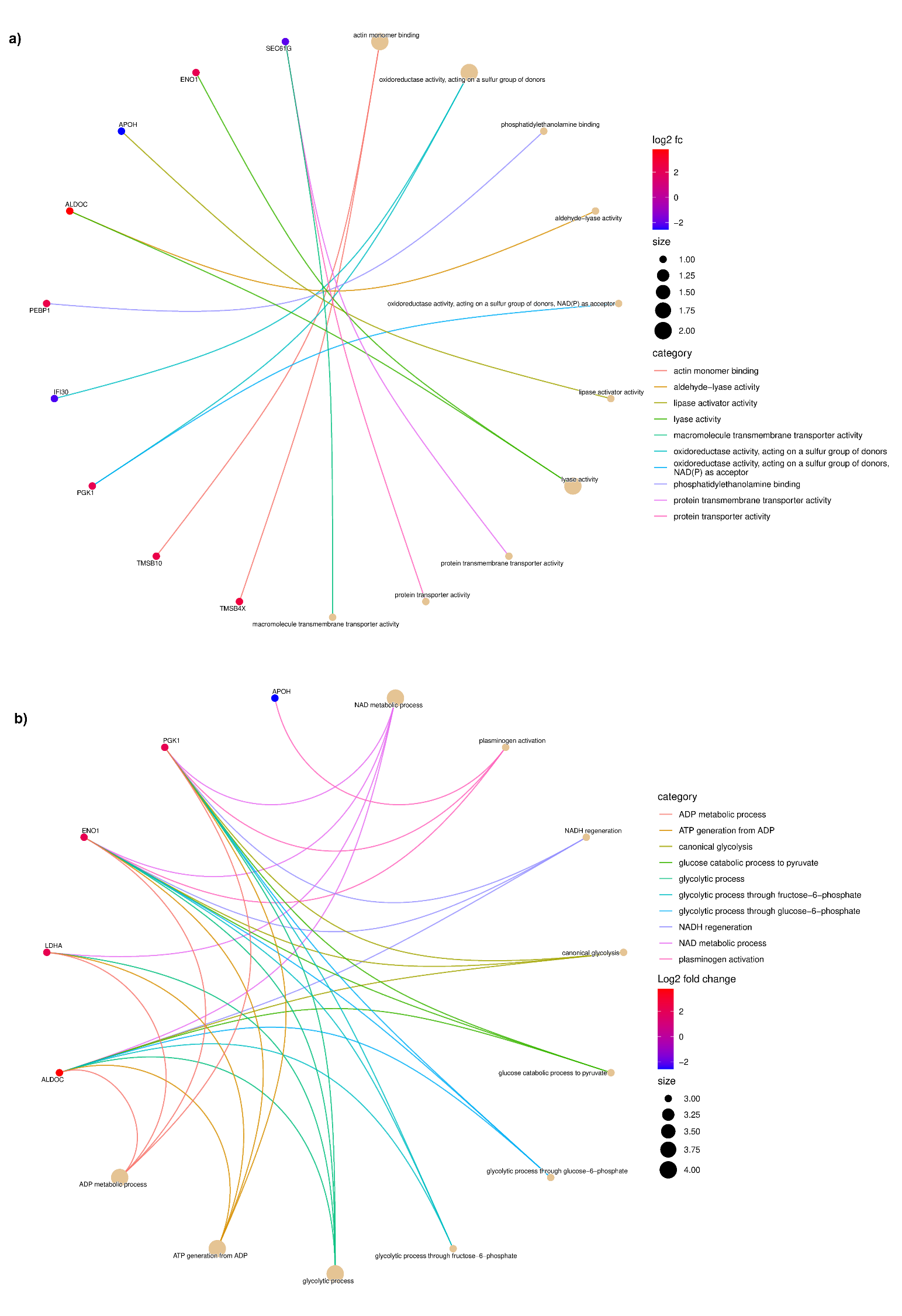


**Supplementary figure 3: Cnet plots for the commonly abundant proteins in the T cells treatment with ADC and SCC-derived sEVs a)** cnetplot of the BP of common DAP **b)** cnetplot of the MF of common DAP.


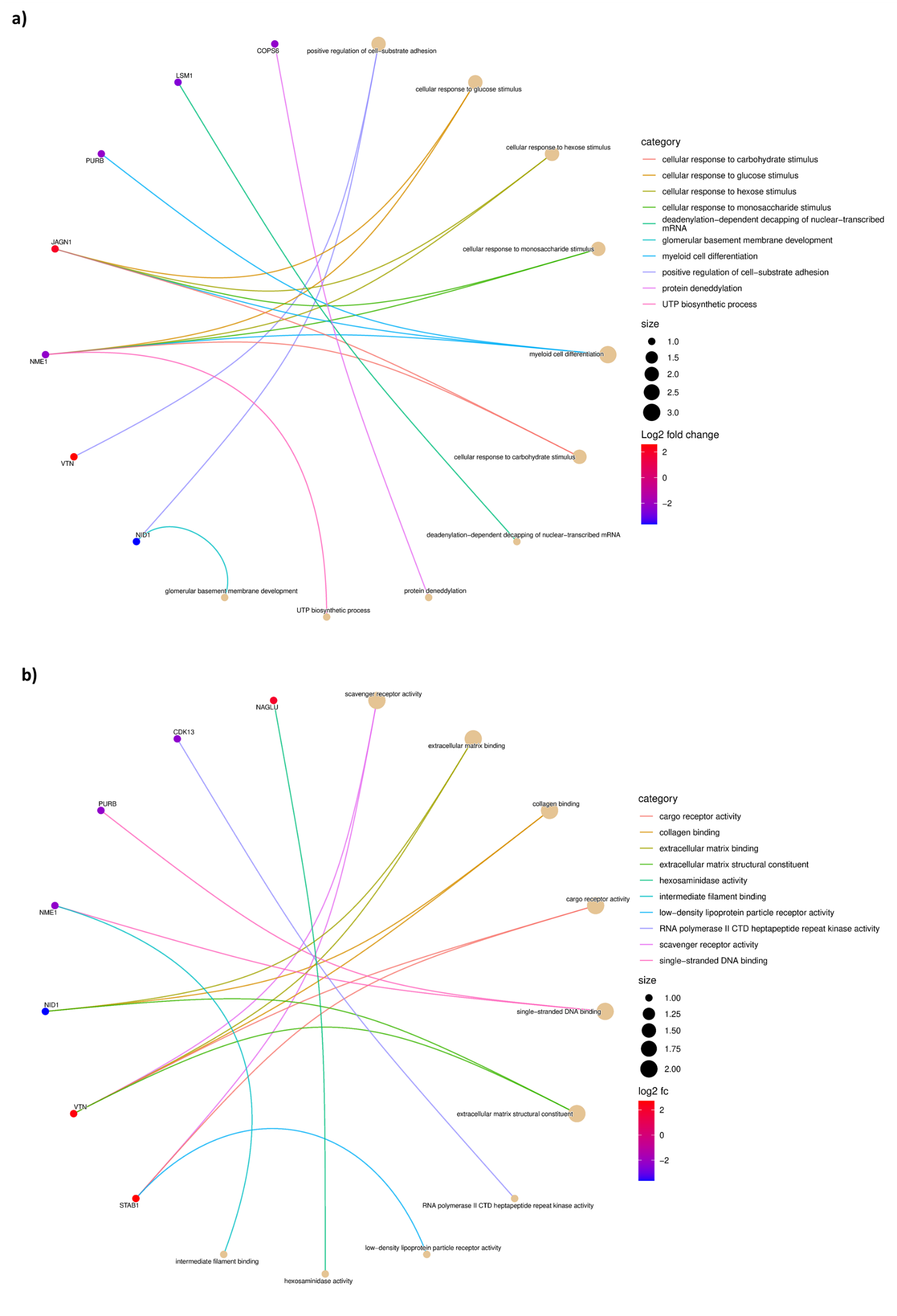


Supplementary figure 4: Involvement of unique differentially abundant proteins (DAP) in the T cells treated with lung ADC-derived exosomes a) cnet plot of biological processes b) cnet plot of molecular function


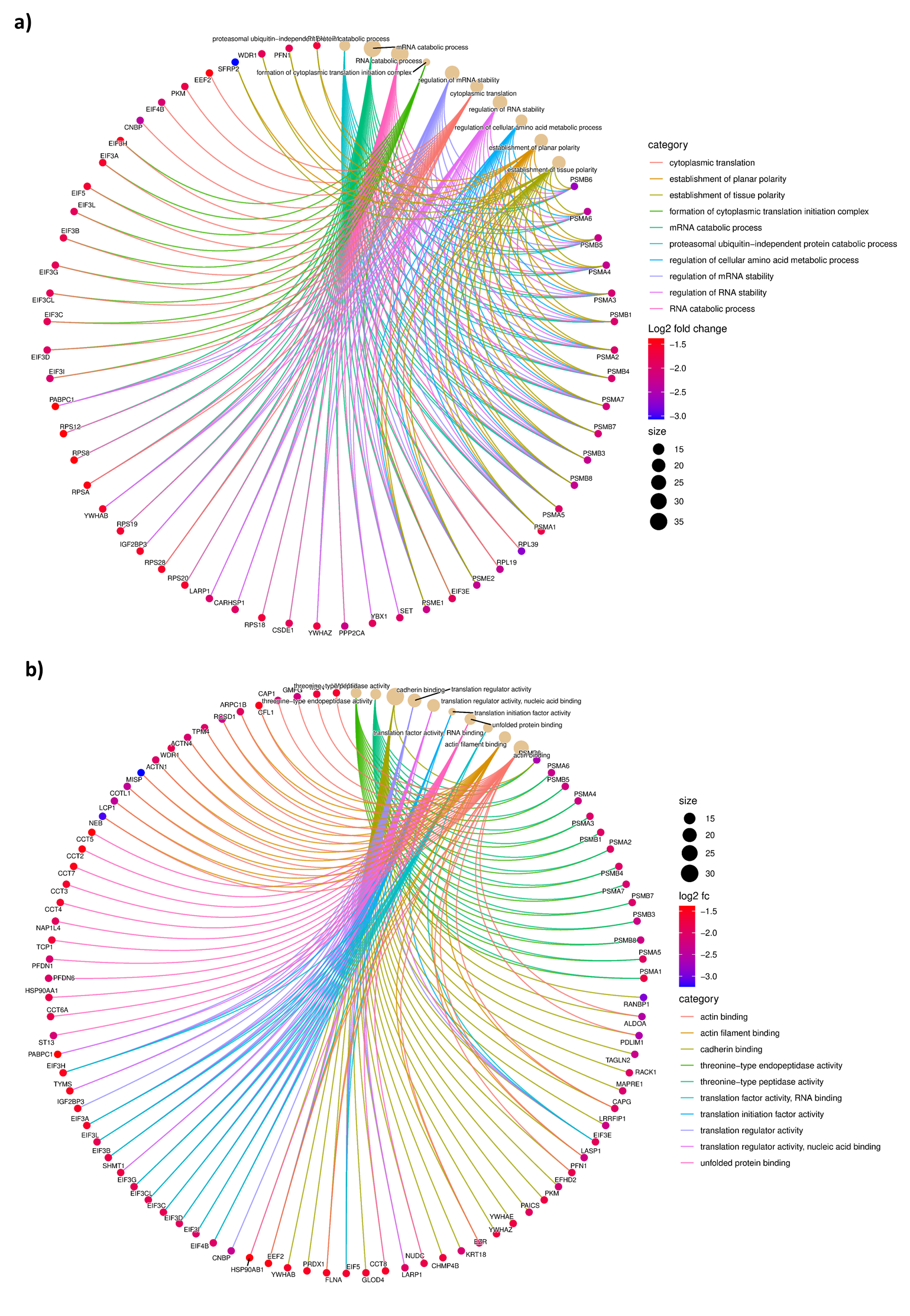


Supplementary figure 5: Involvement of unique DAP in the T cells treated with lung SCC-derived exosomes a) cnet plot of biological processes b) cnet plot of molecular function


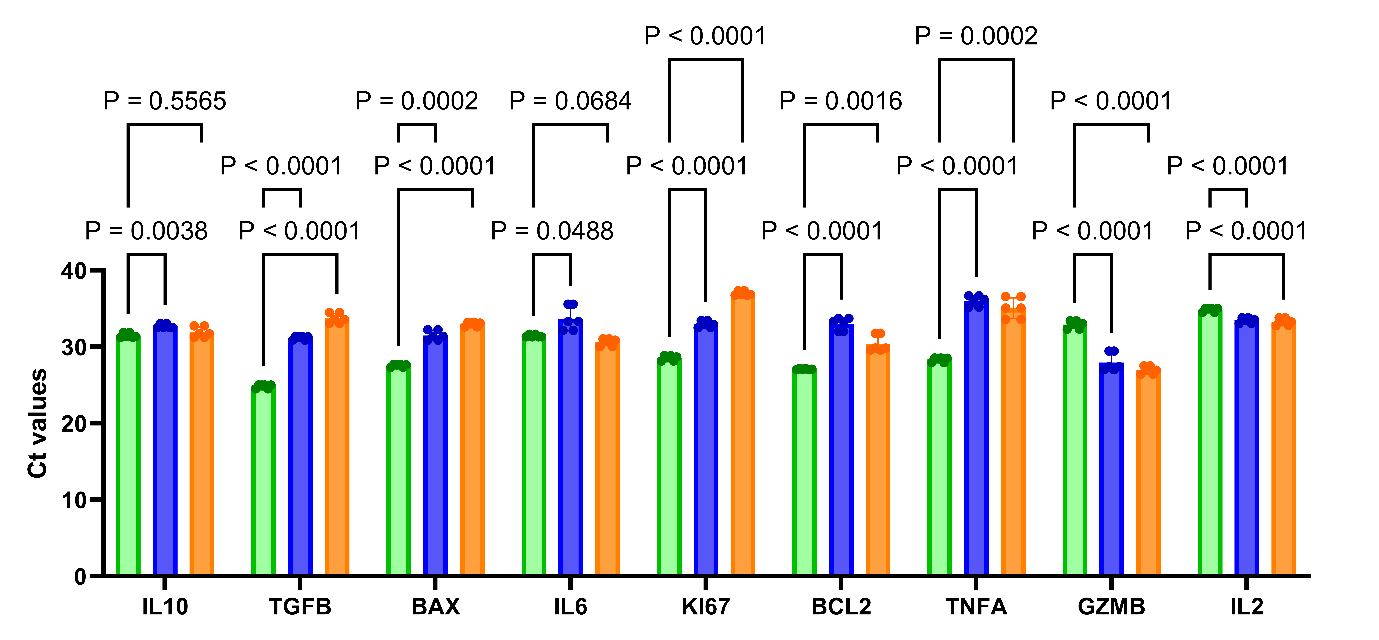


Supplementary figure 6: Ct values of genes that are involved in the immunomodulation of T cells.

Appendix B: Supplementary tables

| Name | Change | Fold change (log2) | Significance |
| --- | --- | --- | --- |
| NID1 | Decreased | -3.634244749 | 4.059424398 |
| KDELR2 | Decreased | -3.473916761 | 3.460920971 |
| DIPK1A | Decreased | -2.734655433 | 2.272767625 |
| ALDOC | Decreased | -2.864136609 | 2.065393997 |
| CCDC25 | Decreased | -2.742221844 | 2.088963362 |
| IGSF9 | Decreased | -2.767231037 | 1.78603672 |
| ANP32A | Decreased | -2.838548442 | 1.668709135 |
| LSM1 | Decreased | -2.584481522 | 1.702989465 |
| JPT1 | Decreased | -2.459431619 | 1.814365197 |
| TMSB4X | Decreased | -2.629939409 | 1.41651985 |
| ZBTB2 | Decreased | -2.517779603 | 1.31609271 |
| COPS6 | Decreased | -2.296604239 | 1.42991116 |
| TMSB10 | Decreased | -2.395611249 | 1.315440728 |
| PEBP1 | Decreased | -2.373230584 | 1.280225044 |
| H2AJ | Decreased | -2.331418598 | 1.290827668 |
| CDK13 | Decreased | -2.3305584 | 1.291505872 |
| NME1 | Decreased | -2.272620455 | 1.054566555 |
| PURB | Decreased | -2.23511432 | 1.070784554 |
| LDHA | Decreased | -2.177279996 | 1.065812331 |
| ENO1 | Decreased | -2.167036722 | 1.051927084 |
| CFAP298 | Decreased | -2.173767068 | 1.031622221 |
| PGK1 | Decreased | -2.154129501 | 1.034628333 |
| STMN1 | Decreased | -2.138486844 | 1.013494893 |
| EXOC3L4 | Decreased | -2.050327554 | 1.088566041 |
| UBE2D3 | Decreased | -2.028215507 | 1.09820264 |
| UFC1 | Decreased | -1.947853143 | 1.001996624 |
| DDI2 | Increased | 2.671163536 | 2.605245825 |
| STAB1 | Increased | 2.717856771 | 2.493727671 |
| SGCD | Increased | 2.708396442 | 2.479827099 |
| PRR22 | Increased | 2.490050854 | 2.072916374 |
| VTN | Increased | 2.590744853 | 1.902679496 |
| APOH | Increased | 2.54793177 | 1.668709135 |
| GPRC5C | Increased | 2.358453971 | 1.766826061 |
| IFI30 | Increased | 2.10780329 | 1.436761971 |
| SEC61G | Increased | 2.083141235 | 1.363219953 |
| GOLGA8R | Increased | 2.070966521 | 1.367420961 |
| NAGLU | Increased | 2.046921047 | 1.11055197 |
| JAGN1 | Increased | 2.011587974 | 1.100526324 |
| CLSTN1 | Increased | 1.98279071 | 1.09820264 |

**Supplementary table 1: DEPs involved in T cells treated with lung adenocarcinoma-derived exosomes with fold change and p value**

| Name | Change | Fold change (log2) | Significance |
| --- | --- | --- | --- |
| SERPINF1 | Increased | 3.307572802 | 7.285370519 |
| SGCD | Increased | 3.943416472 | 6.400848141 |
| APOH | Increased | 2.564904848 | 2.902402993 |
| TMEM258 | Increased | 2.152003093 | 3.076020134 |
| CLSTN1 | Increased | 2.329159664 | 2.341047011 |
| SEC61G | Increased | 2.224317298 | 2.414086121 |
| IFI30 | Increased | 2.251538767 | 2.252823891 |
| P10189 | Increased | 2.10780329 | 2.194958717 |
| CNIH4 | Increased | 2.10159814 | 1.869520608 |
| RFXANK | Increased | 2.089267338 | 1.696432167 |
| GALM | Increased | 2.10780329 | 1.50476496 |
| COX8A | Increased | 1.899695094 | 1.261349931 |
| H3C14 | Increased | 1.652901329 | 1.371581564 |
| H3C15 | Increased | 1.652901329 | 1.371581564 |
| H3C13 | Increased | 1.652901329 | 1.371581564 |
| VMP1 | Increased | 1.816037165 | 1.201417875 |
| NFYA | Increased | 1.756330919 | 1.020315166 |
| PBDC1 | Increased | 1.751465164 | 1.009412511 |
| P4HA2 | Increased | 1.671163536 | 1.029395191 |
| RFX3 | Increased | 1.621488377 | 1.019510214 |
| ALDOC | Decreased | -3.803123688 | 7.521864188 |
| TPI1 | Decreased | -2.885769574 | 5.408159335 |
| MIF | Decreased | -3.164464499 | 5.085148138 |
| ULK4 | Decreased | -3.584240973 | 4.419986555 |
| ENO1 | Decreased | -2.789311926 | 5.032440704 |
| RANBP1 | Decreased | -2.9347052 | 4.735273788 |
| PEBP1 | Decreased | -2.898208353 | 4.546821771 |
| RPL39 | Decreased | -2.726395292 | 4.611380176 |
| NEB | Decreased | -3.143066832 | 4.139198245 |
| EXOC3L4 | Decreased | -3.394925655 | 3.77956144 |
| TMSB4X | Decreased | -2.773996325 | 3.987774856 |
| PSMB6 | Decreased | -2.622227569 | 4.137368939 |
| RRM2 | Decreased | -2.864334741 | 3.825992671 |
| ALDOA | Decreased | -2.542010356 | 4.123193527 |
| LDHA | Decreased | -2.514501036 | 4.026857553 |
| PGK1 | Decreased | -2.512732563 | 4.020959559 |
| STMN1 | Decreased | -2.773363425 | 3.37648551 |
| LCP1 | Decreased | -2.418189948 | 3.698237357 |
| PDCD5 | Decreased | -2.631104282 | 3.472110971 |
| GSTO1 | Decreased | -2.447843644 | 3.617513788 |
| ENO2 | Decreased | -2.534061602 | 3.426175443 |
| TKT | Decreased | -2.377956634 | 3.564931282 |
| DIPK1A | Decreased | -2.943921327 | 2.982730886 |
| COTL1 | Decreased | -2.413052429 | 3.503269876 |
| TBCA | Decreased | -2.595980979 | 3.207891851 |
| TK1 | Decreased | -3.491724851 | 2.298017654 |
| NME2 | Decreased | -2.589044357 | 3.159855629 |
| RABEP1 | Decreased | -2.987138926 | 2.754473725 |
| TSN | Decreased | -2.794727767 | 2.935501927 |
| UBE2L3 | Decreased | -2.541762619 | 3.157944144 |
| PGAM1 | Decreased | -2.524815928 | 3.121821906 |
| P0C7P4 | Decreased | -3.333566851 | 2.223953852 |
| ANP32A | Decreased | -2.443606651 | 3.103106738 |
| ACAT2 | Decreased | -2.331705216 | 3.11880593 |
| AGL | Decreased | -2.324522612 | 3.11880593 |
| WFDC8 | Decreased | -3.388740605 | 2.004588868 |
| PTMA | Decreased | -2.568761783 | 2.661452473 |
| APEH | Decreased | -2.64362534 | 2.564267013 |
| PSMA6 | Decreased | -2.324234562 | 2.806152171 |
| STRAP | Decreased | -2.372952098 | 2.723165832 |
| MOB1B | Decreased | -2.745022149 | 2.329905964 |
| KRT7 | Decreased | -2.738335504 | 2.315855485 |
| MISP | Decreased | -3.241840184 | 1.79017515 |
| PSMB5 | Decreased | -2.217851097 | 2.792975125 |
| RPL19 | Decreased | -2.284810223 | 2.695750122 |
| ZNF146 | Decreased | -3.385569088 | 1.543717489 |
| PDLIM1 | Decreased | -2.579662897 | 2.268779346 |
| GPI | Decreased | -2.314116431 | 2.496694771 |
| TAGLN2 | Decreased | -2.315856015 | 2.474885616 |
| PYGB | Decreased | -2.177917792 | 2.611019792 |
| SFRP2 | Decreased | -3.070045788 | 1.71175647 |
| PSME2 | Decreased | -2.272620455 | 2.506930132 |
| DHPS | Decreased | -3.009167989 | 1.75070806 |
| GDI2 | Decreased | -2.085084679 | 2.659473353 |
| NUDT5 | Decreased | -2.152832405 | 2.544713524 |
| PDCL3 | Decreased | -3.070561067 | 1.604299198 |
| TPP2 | Decreased | -2.331991778 | 2.332380577 |
| PGD | Decreased | -2.250052025 | 2.393694649 |
| ADA | Decreased | -2.27857955 | 2.343550718 |
| ST13 | Decreased | -2.140451477 | 2.478616948 |
| PNP | Decreased | -2.089498151 | 2.481909184 |
| PGM2 | Decreased | -2.516267344 | 2.050325892 |
| PRDX2 | Decreased | -2.178873958 | 2.372192893 |
| PSMA4 | Decreased | -2.260627908 | 2.226342771 |
| FABP5 | Decreased | -2.15704371 | 2.298902621 |
| CNBP | Decreased | -2.364572432 | 2.079211229 |
| GBE1 | Decreased | -2.52682002 | 1.912446392 |
| CACYBP | Decreased | -2.136519533 | 2.274558982 |
| GLUL | Decreased | -2.564622052 | 1.846424578 |
| PSAT1 | Decreased | -2.034215715 | 2.360437247 |
| AHCY | Decreased | -2.148283363 | 2.231493457 |
| C12orf42 | Decreased | -2.952892642 | 1.420922452 |
| RACK1 | Decreased | -1.981487368 | 2.369457467 |
| PSMA3 | Decreased | -2.049282249 | 2.295176882 |
| CLEC16A | Decreased | -2.915138426 | 1.414815975 |
| PSMB1 | Decreased | -2.008630305 | 2.298902621 |
| FDPS | Decreased | -2.337711092 | 1.943798047 |
| MAPRE1 | Decreased | -2.087462841 | 2.182914941 |
| PSMA2 | Decreased | -1.998556583 | 2.229663178 |
| ANP32B | Decreased | -2.131260215 | 2.093397486 |
| SMIM24 | Decreased | -2.922197848 | 1.298894327 |
| CAPG | Decreased | -2.00252251 | 2.198258311 |
| ACTN1 | Decreased | -2.038436182 | 2.132761351 |
| CDC42BPA | Decreased | -2.01649632 | 2.149877083 |
| PSMB4 | Decreased | -2.012568674 | 2.152081795 |
| STEAP3 | Decreased | -2.869476634 | 1.286944468 |
| SUGT1 | Decreased | -2.311793718 | 1.813156119 |
| TMSB10 | Decreased | -2.707082992 | 1.411539149 |
| PSMA7 | Decreased | -2.098621677 | 1.990771534 |
| UBE2N | Decreased | -2.237563718 | 1.811780664 |
| TALDO1 | Decreased | -2.304218969 | 1.740763198 |
| CCDC25 | Decreased | -2.883425316 | 1.161235483 |
| PSMB7 | Decreased | -2.062812492 | 1.965129791 |
| PSMB3 | Decreased | -2.271126827 | 1.756548405 |
| JMJD1C | Decreased | -2.745667602 | 1.277897631 |
| GLRB | Decreased | -2.6617496 | 1.353354492 |
| UROD | Decreased | -2.7322692 | 1.271846955 |
| CLDND1 | Decreased | -1.947105052 | 2.050325892 |
| MDH1 | Decreased | -2.032453537 | 1.964880649 |
| PSMB8 | Decreased | -2.262733813 | 1.728250115 |
| CNDP2 | Decreased | -1.939226578 | 2.03078617 |
| LRRFIP1 | Decreased | -2.272620455 | 1.679312169 |
| AKAP9 | Decreased | -2.565353457 | 1.367527717 |
| WDR1 | Decreased | -1.859969548 | 2.052264363 |
| LINGO1 | Decreased | -2.587364991 | 1.291080874 |
| EIF4B | Decreased | -2.027861775 | 1.830922064 |
| ACTN4 | Decreased | -2.09423607 | 1.756548405 |
| EIF3E | Decreased | -1.901880564 | 1.93551846 |
| CCT6A | Decreased | -1.837539634 | 1.997048352 |
| ARHGDIB | Decreased | -2.035975744 | 1.776807714 |
| LASP1 | Decreased | -2.234194723 | 1.570074949 |
| PFN1 | Decreased | -1.829037689 | 1.974853687 |
| ESD | Decreased | -2.181102551 | 1.609539573 |
| PSME1 | Decreased | -2.200692666 | 1.58069435 |
| SET | Decreased | -1.951214764 | 1.770088764 |
| XPOT | Decreased | -2.492622329 | 1.227736964 |
| TPM4 | Decreased | -2.025737561 | 1.692596522 |
| HSP90AA1 | Decreased | -1.80240004 | 1.9101835 |
| TMEM63B | Decreased | -1.989865623 | 1.717480922 |
| TXNDC17 | Decreased | -2.160274831 | 1.52660505 |
| FNBP1 | Decreased | -2.096261853 | 1.588242711 |
| LDHB | Decreased | -1.791605725 | 1.88256384 |
| EFHD2 | Decreased | -2.131918679 | 1.531135519 |
| ADSL | Decreased | -1.995303605 | 1.649732713 |
| CAP1 | Decreased | -2.144699025 | 1.482702539 |
| PKM | Decreased | -1.766383141 | 1.819534447 |
| PAICS | Decreased | -1.96014098 | 1.620478181 |
| GMFG | Decreased | -2.217851097 | 1.34218186 |
| RCSD1 | Decreased | -2.295135249 | 1.225724654 |
| YWHAE | Decreased | -1.745882689 | 1.770088764 |
| NSFL1C | Decreased | -2.16542962 | 1.34768909 |
| CRIP1 | Decreased | -2.488515009 | 1.019510214 |
| GSTP1 | Decreased | -1.738551687 | 1.754109549 |
| UBA1 | Decreased | -1.732920382 | 1.740763198 |
| PFDN6 | Decreased | -2.033863452 | 1.42712248 |
| DPP3 | Decreased | -2.059355278 | 1.396966333 |
| YBX1 | Decreased | -1.896465616 | 1.550777314 |
| PPP2CA | Decreased | -2.219091058 | 1.221888908 |
| YWHAZ | Decreased | -1.722466024 | 1.717127035 |
| MSN | Decreased | -1.719840555 | 1.71175647 |
| EZR | Decreased | -1.9347052 | 1.487171072 |
| RRM1 | Decreased | -2.068326861 | 1.350233837 |
| DPYSL2 | Decreased | -1.834306703 | 1.570074949 |
| EIF3I | Decreased | -2.013283587 | 1.38953531 |
| PPM1G | Decreased | -1.913798965 | 1.47849611 |
| FKBP4 | Decreased | -1.824156844 | 1.56597742 |
| CCDC9 | Decreased | -2.193140919 | 1.194539745 |
| ARHGDIA | Decreased | -2.010779839 | 1.361998003 |
| GOT1 | Decreased | -2.133234706 | 1.227736964 |
| MTPN | Decreased | -2.058662839 | 1.295835504 |
| PRMT5 | Decreased | -1.771885579 | 1.578049539 |
| PFDN1 | Decreased | -2.096599207 | 1.247686018 |
| KRT18 | Decreased | -2.112366523 | 1.22381607 |
| CSDE1 | Decreased | -1.86393845 | 1.437101605 |
| TCP1 | Decreased | -1.678522677 | 1.613586495 |
| Q9QB97 | Decreased | -1.673556424 | 1.603950082 |
| GRB2 | Decreased | -2.014712352 | 1.261247016 |
| SOD1 | Decreased | -1.809002775 | 1.466821193 |
| LUC7L2 | Decreased | -1.732486293 | 1.539124959 |
| EIF3D | Decreased | -1.941106311 | 1.328799917 |
| EIF3C | Decreased | -1.853197255 | 1.407917839 |
| EIF3CL | Decreased | -1.853197255 | 1.407917839 |
| PGM1 | Decreased | -1.990591873 | 1.263572363 |
| CHMP4B | Decreased | -1.721153887 | 1.531622161 |
| NAP1L4 | Decreased | -1.956428433 | 1.283979791 |
| RPS18 | Decreased | -1.6622055 | 1.578049539 |
| PSMA5 | Decreased | -1.974529312 | 1.265405248 |
| EIF3G | Decreased | -1.870660627 | 1.363836884 |
| ARPC1B | Decreased | -1.963843998 | 1.244436687 |
| HSPA4 | Decreased | -1.766807151 | 1.437101605 |
| PRDX4 | Decreased | -1.929412288 | 1.266749254 |
| P03198 | Decreased | -2.157690514 | 1.019510214 |
| PDCD4 | Decreased | -2.08983709 | 1.087170527 |
| CAND1 | Decreased | -1.759581973 | 1.414741271 |
| UFC1 | Decreased | -2.089159132 | 1.062261555 |
| NHERF1 | Decreased | -1.688404188 | 1.455621947 |
| PFAS | Decreased | -1.691087441 | 1.445795642 |
| SHMT1 | Decreased | -1.767654798 | 1.366722948 |
| XPNPEP1 | Decreased | -1.882447416 | 1.244736024 |
| NASP | Decreased | -1.753604536 | 1.364942642 |
| PGLS | Decreased | -1.770194755 | 1.336233295 |
| SH3BGRL | Decreased | -1.993493221 | 1.108381288 |
| CCT4 | Decreased | -1.617768646 | 1.482702539 |
| BTF3 | Decreased | -1.690640579 | 1.408821337 |
| JPT1 | Decreased | -2.052415894 | 1.034294054 |
| FAM120A | Decreased | -1.803640357 | 1.28195948 |
| SRSF6 | Decreased | -1.793687837 | 1.291464543 |
| EIF3B | Decreased | -1.783666181 | 1.294776227 |
| CARHSP1 | Decreased | -1.930548119 | 1.142913021 |
| NAP1L1 | Decreased | -1.754887502 | 1.314832509 |
| OXSR1 | Decreased | -1.769348599 | 1.294776227 |
| CCT3 | Decreased | -1.604545816 | 1.453111307 |
| GLO1 | Decreased | -2.011853406 | 1.039617597 |
| NUDC | Decreased | -1.800330473 | 1.247768704 |
| EIF3L | Decreased | -1.874206786 | 1.168896062 |
| LARP1 | Decreased | -1.987684678 | 1.047238718 |
| PPA1 | Decreased | -1.709731759 | 1.319050329 |
| FKBP1A | Decreased | -1.997111721 | 1.013602837 |
| UFM1 | Decreased | -1.890252115 | 1.114450592 |
| LANCL1 | Decreased | -1.9800253 | 1.009412511 |
| FKBP3 | Decreased | -1.708849377 | 1.272631454 |
| PAFAH1B1 | Decreased | -1.667210912 | 1.295198732 |
| THBS1 | Decreased | -1.943358763 | 1.007932562 |
| CCT8 | Decreased | -1.570462931 | 1.380598368 |
| CLTA | Decreased | -1.727267011 | 1.221888908 |
| GLOD4 | Decreased | -1.640158155 | 1.306761535 |
| UBE2V1 | Decreased | -1.929790998 | 1.010286113 |
| PEDS1-UBE2V1 | Decreased | -1.929790998 | 1.010286113 |
| YWHAH | Decreased | -1.606915942 | 1.326781453 |
| ACOT7 | Decreased | -1.863542051 | 1.069832818 |
| HINT1 | Decreased | -1.714575239 | 1.19601063 |
| EIF5 | Decreased | -1.61022759 | 1.289251851 |
| GRK2 | Decreased | -1.628540319 | 1.259887248 |
| SUMO3 | Decreased | -1.831877241 | 1.054481377 |
| FLNA | Decreased | -1.544485386 | 1.329353867 |
| SORD | Decreased | -1.855989697 | 1.011453649 |
| PSMA1 | Decreased | -1.771463058 | 1.081135295 |
| PRDX1 | Decreased | -1.537047519 | 1.314832509 |
| ACTR1A | Decreased | -1.649845352 | 1.194702533 |
| COX6B1 | Decreased | -1.655351829 | 1.162413487 |
| SRSF2 | Decreased | -1.702657543 | 1.111071342 |
| ACP1 | Decreased | -1.710613602 | 1.099900485 |
| RPS20 | Decreased | -1.597411988 | 1.1924536 |
| NCL | Decreased | -1.508935662 | 1.26288667 |
| GLRX3 | Decreased | -1.561692721 | 1.193802685 |
| YWHAG | Decreased | -1.662661255 | 1.08935692 |
| CFL1 | Decreased | -1.496717988 | 1.240501848 |
| SKIC8 | Decreased | -1.649385529 | 1.062261555 |
| RPS28 | Decreased | -1.55924706 | 1.15004385 |
| SNRPD2 | Decreased | -1.572889668 | 1.118587649 |
| EIF3A | Decreased | -1.579180148 | 1.101525731 |
| IGF2BP3 | Decreased | -1.546956178 | 1.130493586 |
| CCT7 | Decreased | -1.473007568 | 1.194888278 |
| STIP1 | Decreased | -1.62058641 | 1.038617524 |
| MAPK1 | Decreased | -1.567058626 | 1.07996764 |
| RPS19 | Decreased | -1.622461867 | 1.021892313 |
| YWHAB | Decreased | -1.578214165 | 1.062261555 |
| CHORDC1 | Decreased | -1.541019153 | 1.094407408 |
| SNRPD1 | Decreased | -1.617298483 | 1.016277932 |
| CDV3 | Decreased | -1.509442493 | 1.117775151 |
| RPSA | Decreased | -1.45206823 | 1.153595182 |
| COX17 | Decreased | -1.534061602 | 1.051243003 |
| EEF2 | Decreased | -1.443076151 | 1.136088824 |
| TYMS | Decreased | -1.510961919 | 1.063941417 |
| CCT2 | Decreased | -1.44148348 | 1.132795278 |
| EIF3H | Decreased | -1.515005916 | 1.05294276 |
| PRKRA | Decreased | -1.516015147 | 1.034518287 |
| CAPRIN1 | Decreased | -1.511973982 | 1.024316317 |
| HSP90AB1 | Decreased | -1.425459305 | 1.101730371 |
| TUBA1C | Decreased | -1.424922088 | 1.101588987 |
| RPS8 | Decreased | -1.420617139 | 1.094407408 |
| PAFAH1B3 | Decreased | -1.489542936 | 1.021494863 |
| CCT5 | Decreased | -1.412510571 | 1.07996764 |
| RPS12 | Decreased | -1.407080775 | 1.069311409 |
| SEC24C | Decreased | -1.462575888 | 1.000832363 |
| PABPC1 | Decreased | -1.378511623 | 1.019510214 |

**Supplementary table 2: DEPs involved in T cells treated with lung squamous cell carcinoma-derived exosomes group with fold change and p value**
