# Supplementary material for: Small Extracellular Vesicle (sEV) Uptake from Lung Adenocarcinoma and Squamous Cell Carcinoma Alters T-Cell Cytokine Expression and Modulates Protein Profiles in sEV Biogenesis
Source: Proteomes. 2025 Apr 23;13(2):15. doi: 10.3390/proteomes13020015 (PMC12101295; doi:10.3390/proteomes13020015)
Supplement: Supplementary file 1 [file proteomes-13-00015-s001.zip › proteomes supplementary/raw images for publication.pptx]

## Slide 1
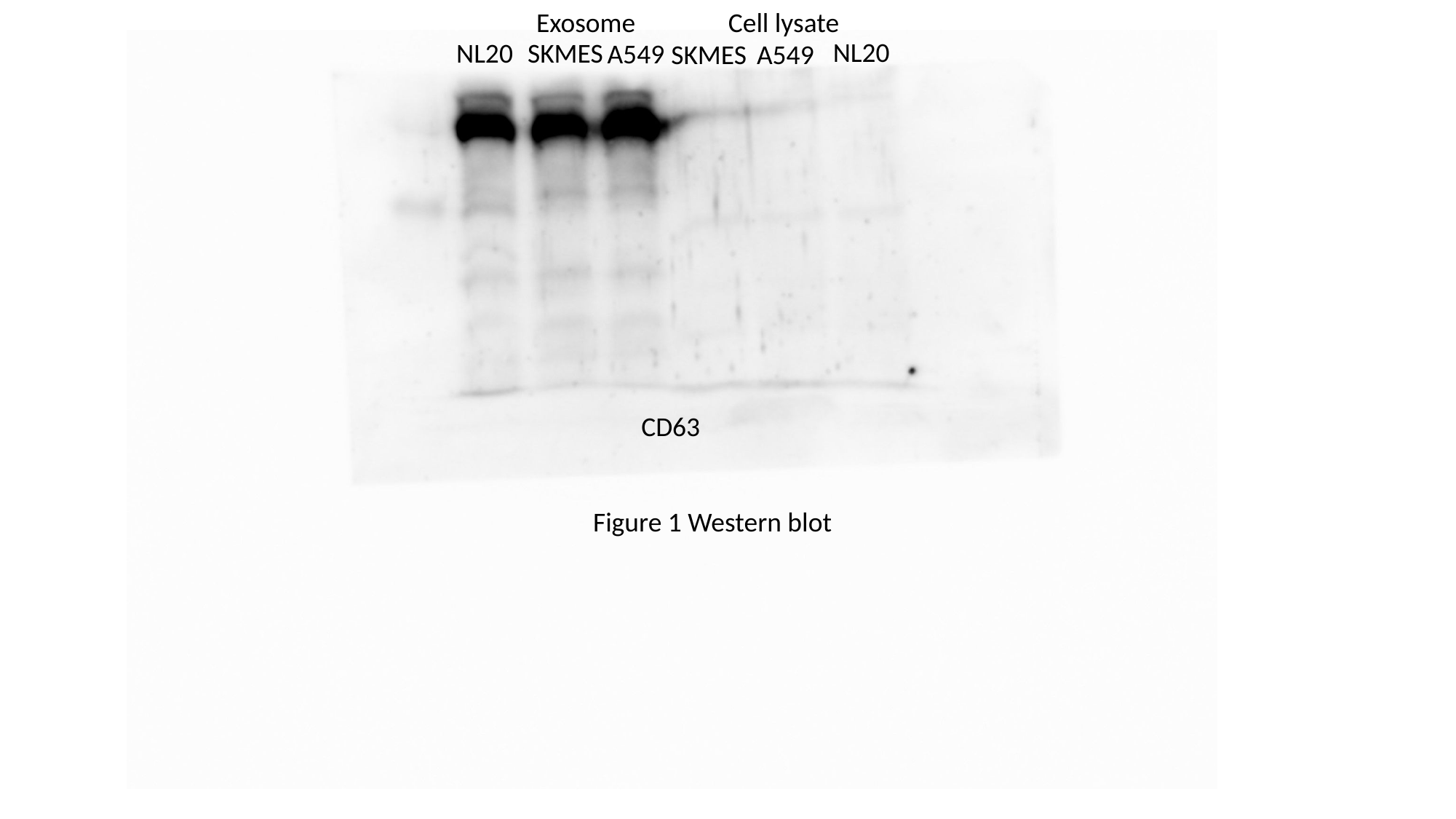

Exosome
Cell lysate
NL20
NL20
SKMES
A549
A549
SKMES
CD63
Figure 1 Western blot

## Slide 2
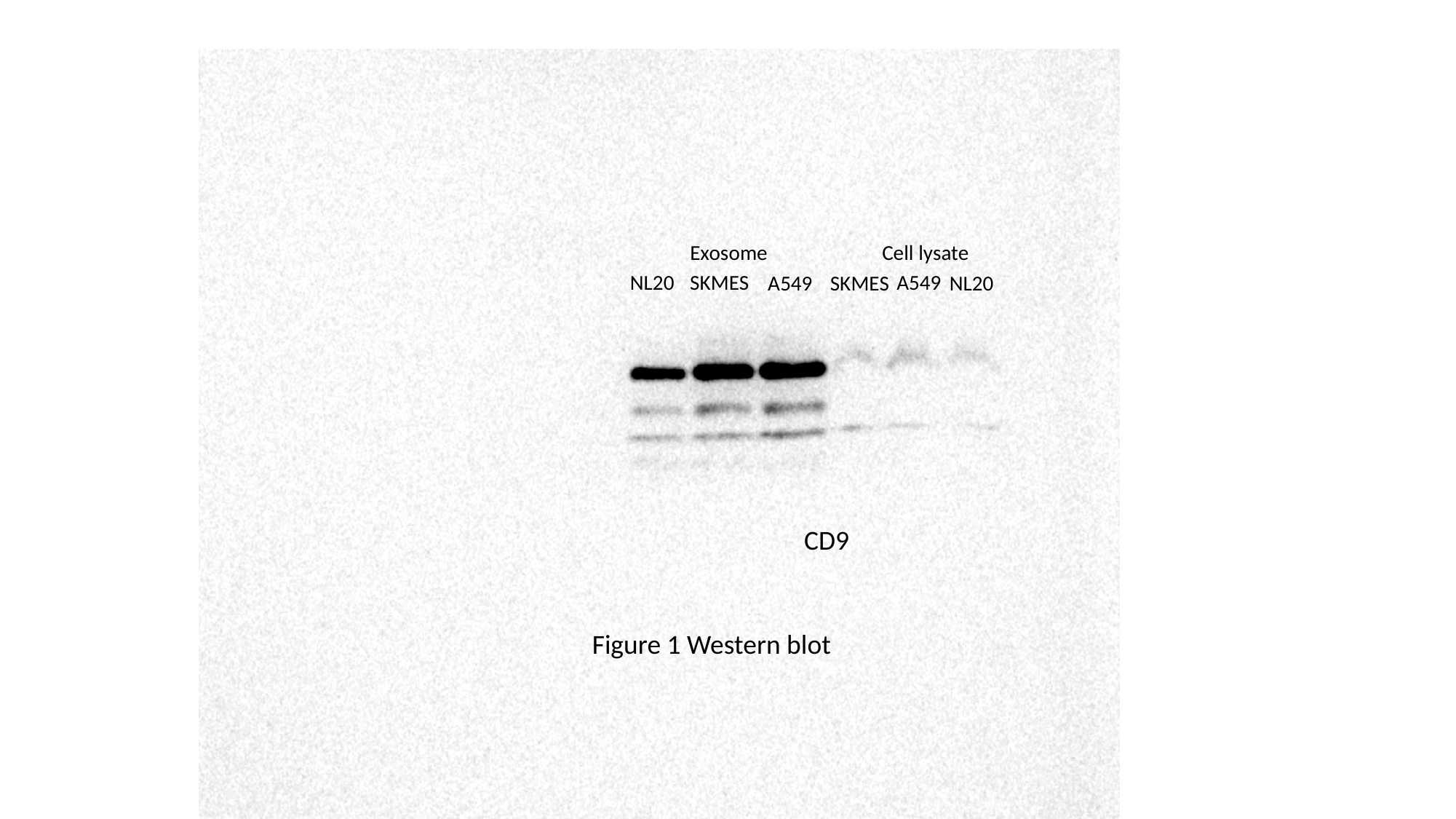

Exosome
Cell lysate
NL20
SKMES
A549
SKMES
A549
NL20
CD9
Figure 1 Western blot

## Slide 3
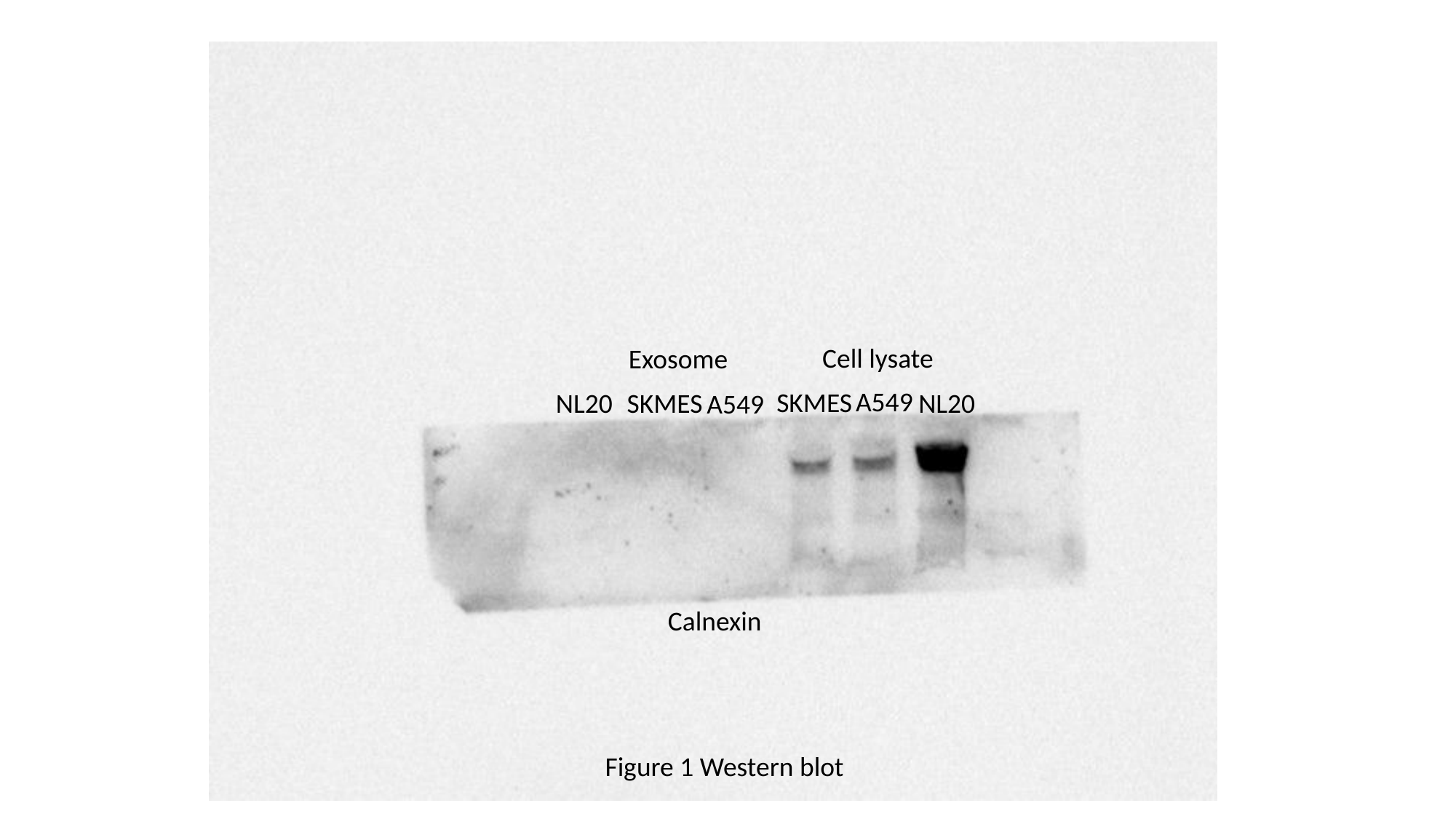

Cell lysate
Exosome
A549
SKMES
NL20
SKMES
NL20
A549
Calnexin
Figure 1 Western blot

## Slide 4
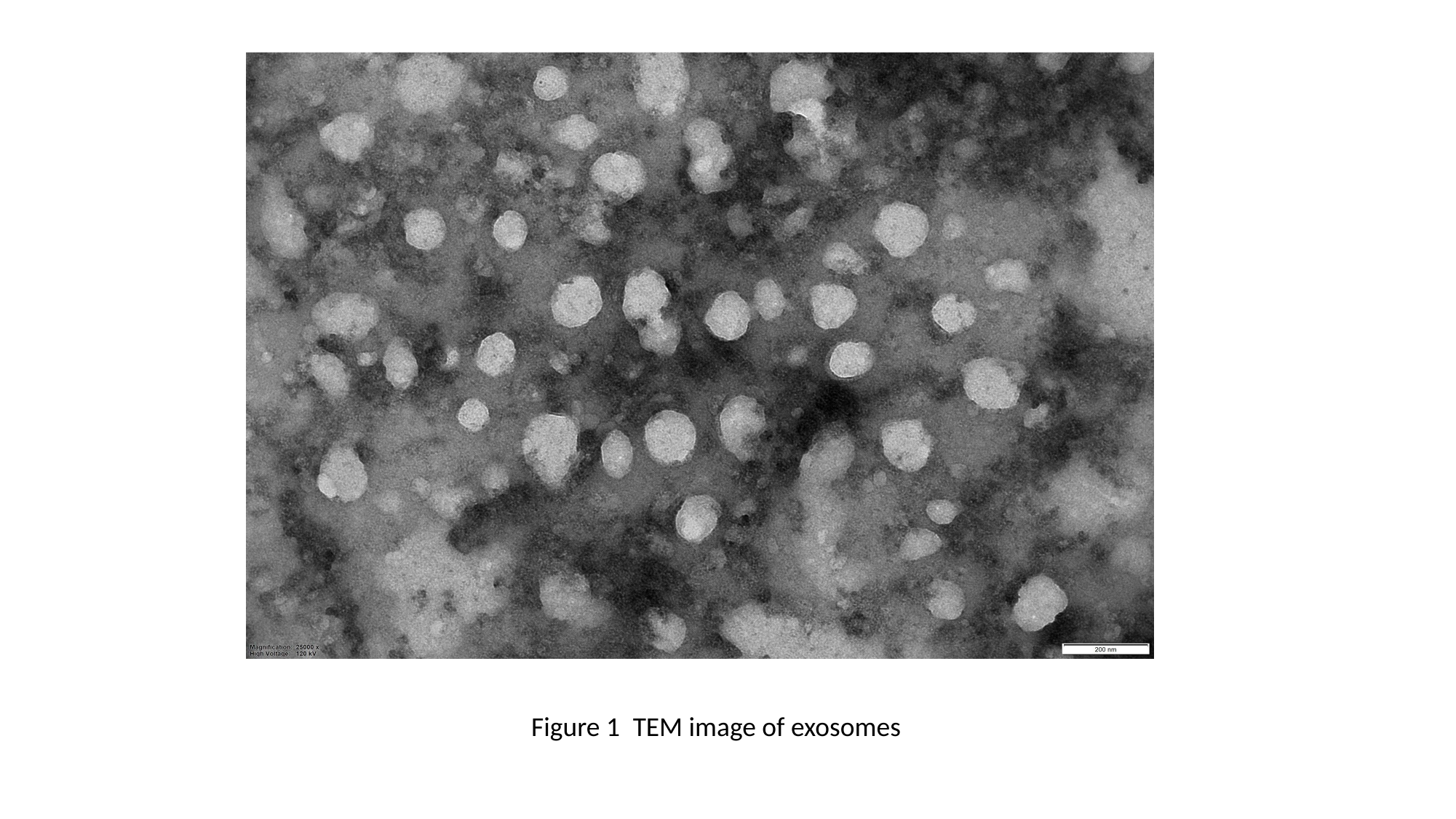

Figure 1
TEM image of exosomes

## Slide 5
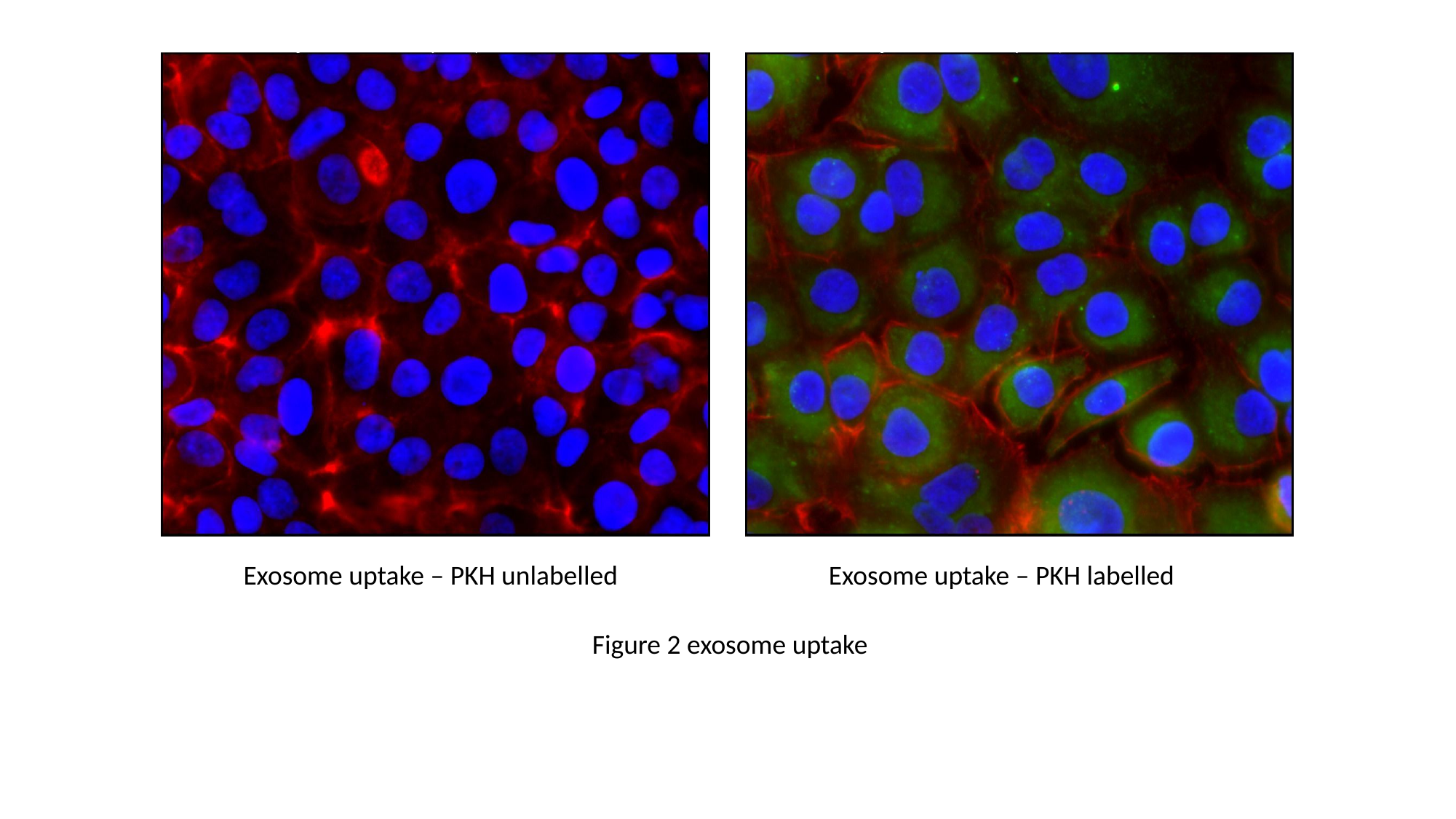

Exosome uptake – PKH unlabelled
Exosome uptake – PKH labelled
Figure 2 exosome uptake
